# Quantifying trends and uncertainty in prehistoric forest composition in the upper Midwestern United States

**DOI:** 10.1002/ecy.2856

**Published:** 2019-09-13

**Authors:** Andria Dawson, Christopher J. Paciorek, Simon J. Goring, Stephen T. Jackson, Jason S. McLachlan, John W. Williams

**Affiliations:** ^1^ Department of General Education Mount Royal University Calgary Alberta T3E6K6 Canada; ^2^ Department of Statistics University of California Berkeley California 94720 USA; ^3^ Department of Geography and Center for Climatic Research University of Wisconsin‐Madison Madison Wisconsin 53706 USA; ^4^ Department of the Interior Southwest Climate Science Center U.S. Geological Survey Tucson Arizona 85721 USA; ^5^ Department of Geosciences University of Arizona Tucson Arizona 85721 USA; ^6^ Department of Biological Sciences University of Notre Dame Notre Dame Indiana 46556 USA

**Keywords:** Bayesian hierarchical models, forest dynamics, historical ecology, paleoecology, palynology, pollen–vegetation modeling, *Tsuga*

## Abstract

Forest ecosystems in eastern North America have been in flux for the last several thousand years, well before Euro‐American land clearance and the 20th‐century onset of anthropogenic climate change. However, the magnitude and uncertainty of prehistoric vegetation change have been difficult to quantify because of the multiple ecological, dispersal, and sedimentary processes that govern the relationship between forest composition and fossil pollen assemblages. Here we extend STEPPS, a Bayesian hierarchical spatiotemporal pollen–vegetation model, to estimate changes in forest composition in the upper Midwestern United States from about 2,100 to 300 yr ago. Using this approach, we find evidence for large changes in the relative abundance of some species, and significant changes in community composition. However, these changes took place against a regional background of changes that were small in magnitude or not statistically significant, suggesting complexity in the spatiotemporal patterns of forest dynamics. The single largest change is the infilling of *Tsuga canadensis* in northern Wisconsin over the past 2,000 yr. Despite range infilling, the range limit of *T. canadensis* was largely stable, with modest expansion westward. The regional ecotone between temperate hardwood forests and northern mixed hardwood/conifer forests shifted southwestward by 15–20 km in Minnesota and northwestern Wisconsin. *Fraxinus*,* Ulmus*, and other mesic hardwoods expanded in the Big Woods region of southern Minnesota. The increasing density of paleoecological data networks and advances in statistical modeling approaches now enables the confident detection of subtle but significant changes in forest composition over the last 2,000 yr.

## Introduction

The presettlement data can be interpreted as a stable baseline and used to evaluate changes in the landscape caused by humans. Such an evaluation is possible because the geographic distributions of major tree species…have changed little over the last 3000 years. (Frelich 1995)

Estimating the population and community dynamics of trees in the millennia before the rapid changes of the past two centuries is important for conservation biology and global‐change ecology. The rates and patterns of vegetation change preceding agroindustrial society offer baseline targets for management of natural areas (Barnosky et al. 2017) and benchmarks for assessing subsequent change (National Research Council 2005, Willard and Bernhardt 2011, Jackson 2012). Long‐term ecological time series provide constraints on processes in tree communities that play out over centennial to millennial timescales, that is, at timescales beyond human perception and the instrumental record (Magnuson 1990). Records of past forest dynamics are also helpful for assessing the sensitivity of forests to past environmental change (Nolan et al. 2018) and the degree of disequilibrium between climate change and forest response (Svenning and Sandel 2013, Blonder et al. 2018). Because current accelerated rates of environmental change are expected to persist indefinitely, extending our understanding of forest dynamics across a range of timescales is essential to forecast future forest dynamics (Dietze 2017).

Networks of fossil pollen data provide empirical evidence about dynamics of plant populations and communities at timescales of decades to millennia. Fossil pollen data have provided rough estimates of range expansions and contractions (Woods and Davis 1989, Davis et al. 1991, Parshall 2002), continental‐scale changes in taxon distributions (Bernabo and Webb 1977, Williams et al. 2004), and local‐ to landscape‐scale community shifts (Grimm 1983, Umbanhowar et al. 2006, Hotchkiss et al. 2007, Jackson et al. 2014) driven by long‐term changes in climate and disturbance regimes. However, quantifying past trends in forest composition with uncertainty has been more difficult. Despite a century of pollen analysis (Edwards et al. 2017), obtaining robust statistical estimates of the rates and patterns of past forest change remains a major research frontier.

The key challenge for deriving robust inference about the rates and patterns of population and community change in forests from fossil pollen data is that the relationship between the data (changing relative abundances of pollen types in sediment cores from networks of lakes or mires) and the subject of interest (changing abundances of taxa across a landscape) is noisy and governed by many interacting processes (Webb and McAndrews 1976, Webb et al. 1993, Jackson 1994). Differential pollen production and dispersal, long‐distance pollen transport and deposition, and uncertainty in age estimates all contribute to uncertainty in inference about the changing abundance of trees on the landscape. Additionally, differences in the spatial and temporal scales of pollen and forest survey data must be reconciled. Pollen records integrate pollen source radii ranging from tens of meters to hundreds of kilometers (Bradshaw and Webb 1985, Prentice et al. 1987, Jackson 1994, Calcote 1995), depending on species and the size and type of sedimentary basin, and they typically have decadal‐ to centennial‐scale resolution because of sediment mixing, sampling density, and decadal‐ to centennial‐scale uncertainties in age inferences (Webb 1993, Goring et al. 2012, Liu et al. 2012). Conversely, forest survey data in the eastern United States are typically collected at the scale of the individual tree for individual survey points or plots (Schulte and Mladenoff 2001) and can then be aggregated to study vegetation patterns at broader spatial grains and extents. Individual surveys represent discrete snapshots in time that are sometimes repeated (Woudenberg et al. 2011), and sometimes not (Goring et al. 2016).

Disentangling these processes and scale interactions is not easy. Much of paleoecology's contribution to conservation biology and global‐change ecology has consequently relied on analyses of raw pollen abundance data (Hunter et al. 1988, Williams et al. 2013, Maguire et al. 2016). Other efforts have sought to build quantitative and process‐based models for inferring vegetation composition and structure from fossil pollen (Prentice 1985, Sugita 2007a, b, Gaillard et al. 2010, Williams et al. 2011, Mazier et al. 2015). However, even these quantitative reconstructions usually do not provide statistically robust estimates of uncertainty.

The upper Midwestern United States (UMW), ranging from Minnesota to Upper Michigan, has been the focus of decades of paleoecological study and has one of the densest networks of fossil pollen data worldwide. Key ecological changes include the ongoing westward range expansion of several tree taxa in the Great Lakes region (Davis et al. 1986, Woods and Davis 1989, Jackson and Booth 2002, Booth et al. 2012a, Jackson et al. 2014, Wang et al. 2016), population expansion for some species such as hemlock (Davis et al. 1998) and white pine (Tweiten et al. 2015), the establishment of mesic hardwood forests in the Big Woods of south‐central Minnesota (Umbanhowar et al. 2006, Shuman et al. 2009, Hupy 2012), and southward shifts in the ecotone between northern mixed forests and temperate broadleaved forests (Hupy 2012). There is also evidence for ecologically significant climatic changes in the UMW during the past 3,000 yr (Booth et al. 2002, 2006, Booth and Jackson 2003, Shuman et al. 2009, Tweiten et al. 2009) and shifts in fire regimes (Umbanhowar 2004).

Qualitatively, these forest compositional changes are clear across fossil pollen records. However, they are small relative to those associated with the last deglaciation and Euro‐American land use (Frelich 1995), and interpretations of raw pollen percentage data present problems typical of paleoecological interpretation. Given the small magnitude of some reported trends over the last 2,000 yr and uncertainty in observations, are these changes truly significant, statistically or ecologically? Can the magnitude of past compositional changes be determined, with quantified uncertainty?

Here, we use the hierarchical Bayesian model STEPPS (Dawson et al. 2016) to predict the changes in the relative abundance of tree taxa over the last two millennia. STEPPS reconstructs spatiotemporal variations in forest composition from fossil pollen data, using a process‐based statistical representation of pollen productivity and dispersal. STEPPS assesses uncertainty in data and parameters and produces gridded probabilistic estimates of tree abundance. These posterior estimates make it possible to assess changes (and associated uncertainty) at multiple levels of ecological organization, ranging from individual taxa to community‐level indices of change. We take the epigraph from Frelich as our challenge: Using an exceptionally dense paleoecological network and modern statistical techniques, we ask whether systematic community changes in the millennia preceding agroindustrial society are large enough to rise above statistical noise. We further ask: Were such changes uniform across the landscape, or were they patchy in space and/or time? What were the rates of shifts in populations, communities, and ecotones? Where is there statistically significant evidence for either change or stability?

## Data

**Figure 1:**
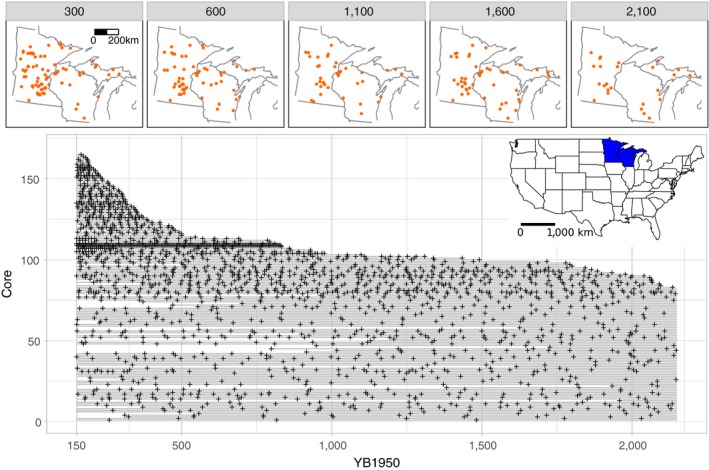
Upper: Availability of pollen records for some of the considered time intervals. Lower: Time span of the pollen records from 2,150 to 150 YB1950 (light gray lines) and the intervals with pollen samples (black crosses) for each record. Dates represent mean estimates from the Bacon‐generated age posteriors. Lower inset: Map of the United States highlighting the study area.

**Figure 2:**
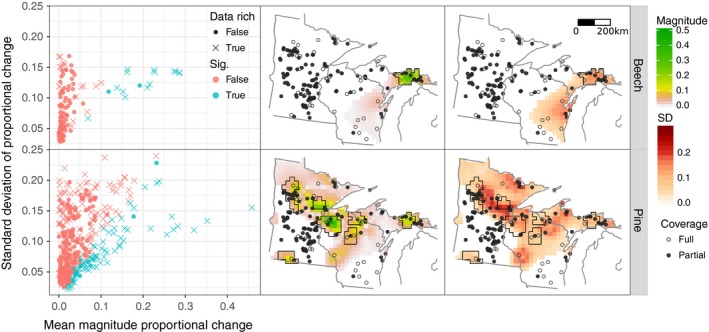
Summary of the means and standard deviations of posterior change of relative abundance of beech (top row) and pine (bottom row) between 2,100 and 300 YB1950. Left: Scatter plot of the posterior means and standard deviations of the changes in proportions. Statistical significance of the change distinguished by color. Sites informed by more than one pollen record in a 50 km radius are indicated by crosses, and sites with one or zero sites within 50‐km indicated by points. Center: Absolute value of the posterior mean magnitude of change. Right: Posterior standard deviation of the magnitude of change. Both center and right: Outlined regions indicate areas that underwent statistically significant change.

We focus on a part of the UMW that comprises Minnesota, Wisconsin, and the Upper Peninsula of Michigan. We choose this spatial domain for three reasons: (1) the dense network of records allows for a regional‐scale reconstruction (Fig. 1), (2) the area contains several major climatically sensitive ecotones, and (3) the well‐established paleoecological literature provides a series of reported vegetation changes that can be tested.

As in Dawson et al. (2016), we focus on 10 tree genera that include the most abundant taxa as well as several taxa that are less abundant but have been hypothesized in the literature to have undergone range shifts or compositional changes in the UMW over the recent millennia: *Acer* (maple), *Betula* (birch), *Fagus* (beech), *Fraxinus* (ash), *Larix* (tamarack), *Picea* (spruce), *Pinus* (pine), *Quercus* (oak), *Tsuga* (hemlock), and *Ulmus* (elm). The remaining tree taxa “Other Hardwood” and “Other Conifer” represent an aggregation of the remaining hardwood and conifer taxa.

### Pollen records

Sediment pollen records were obtained from the Neotoma Paleoecology Database (Williams et al. 2018) using the R *neotoma* package (Goring et al. 2015). Each record includes pollen counts for multiple depths in a sediment core, usually from a lake or mire, and a set of age controls used to constrain age‐depth models.

Inferring temporal changes from the pollen records requires that each pollen sample be associated with an age estimate or age distribution. In an effort toward consistency and uncertainty quantification, age‐depth models were refit for all pollen records in the spatial domain using Bacon (Blaauw and Christen 2011). Age‐depth model construction methods and results are described in more detail in Goring et al. (2019) and Kujawa et al. (2016). We follow radiocarbon and paleoecological convention of representing time as “years before present,” where present is fixed at the year 1950 (Vogel 1969). We use this time scale here, and denote its use by YB1950.

The pollen records cover the spatial domain, although the sampling density in Minnesota is much higher than in Wisconsin and the Upper Peninsula of Michigan (Fig. 1), largely due to work by Edward J. Cushing (University of Minnesota, *unpublished data*). In this work we consider the two millennia preceding Euro‐American settlement (2,150–150 YB1950). Fifty‐six pollen records span the entire temporal domain of interest, and another 147 records cover a portion of this interval (Fig. 1).

### Calibration data set

As described in (Dawson et al. 2016), Public Land Survey (PLS) forest composition data compiled by Goring et al. (2016) and statistically interpolated by Paciorek et al. (2016), as well as settlement‐era pollen data, were used to calibrate STEPPS. The PLS compilation for this domain comprised 367,209 corner points and included various corrections to minimize surveyor biases in PLS data, including spatially varying correction factors for sampling design, corrections for azimuthal censoring, setting minimum diameter limits, aggregation to genus, and aggregation to an 8‐km grid (Goring et al. 2016, Cogbill et al. 2018). This 8‐km grid defines the spatial resolution of the STEPPS calibration model.

## Methods

STEPPS is used to estimate relative abundance, defined to be the proportion of total stems represented by each species; we refer to this reconstructed variable as relative abundance or forest composition. In this application of the model, relative abundance is defined by the statistically interpolated PLS tree count data (Paciorek et al. 2016); which represents trees with a diameter greater than 8 inches at 1.4 m (diameter at breast height, dbh). In the STEPPS modeling framework, there are two stages to estimating past forest composition: calibration and prediction. The calibration stage requires the quantification, with uncertainty, of key process parameters, including pollen productivity and dispersal. The prediction stage uses the calibration parameter estimates in conjunction with fossil pollen samples to infer past forest composition. The prediction is done in a way that borrows strength across space and time; in other words, the model accounts for the spatial and temporal dependence of forest composition. Here we focus on the methods and models used in the prediction stage, which build upon previous work presented in Paciorek and McLachlan (2009) and Dawson et al. (2016).

### Calibration stage

The pollen–vegetation calibration model defines the theoretical relationship between pollen and vegetation. Pollen counts at sedimentary basin *i* for taxon *k* are denoted by *y*_*i*,*k*_. Pollen counts are modeled by a Dirichlet‐multinomial distribution to account for statistical overdispersion; see Dawson et al. (2016). So,

$$y_{i,\cdot }∼\text{DM}\left(n_{i},\varphi_{i}\right)$$

where $n_{i}=\sum_{k=1}^{K}y_{i,k}$. The sum $\sum_{i}\varphi_{i}$ quantifies the degree of overdispersion relative to the multinomial distribution and is equal to the sum of the local and nonlocal pollen contributions of forests in the spatial domain. For location *i* located within grid cell *s*(*i*), we denote the forest composition of taxon *k* by *r*_*s*(*i*),*k*_. Then

(1) $$\varphi_{i,k}=\gamma_{k}\phi_{k}r_{s(i),k}+\frac{1}{C}\left(1-\gamma_{k}\right)\phi_{k}\sum_{s_{j}\neq s\left(i\right)}r_{s(i),k}w_{k}\left(s\left(i\right),s_{j},\theta \right)$$

where γ_*k*_ represents the relative contributions of local and nonlocal pollen for taxon *k*, ϕ_*k*_ represents pollen production for taxon *k*,* w*_*k*_(*s*(*i*), *s*_*j*_, **θ**) quantifies the weights of the relative contribution of pollen from cell *s*_*j*_ to focal cell *s*(*i*) as a function of parameters in the weight function θ. Finally, *C* represents a scaling factor equal to the sum of the weights in the spatial domain.

The values of the weights *w*_*k*_(*s*(*i*), *s*_*j*_, **θ**) are determined by a specified parametric dispersal kernel, the parameters of which are estimated during model fitting. Here we use the symmetric and spatially invariant inverse power‐law kernel (Austerlitz et al. 2004); this kernel had a better predictive ability than the shorter‐tailed Gaussian kernel (Dawson et al. 2016). Posterior estimates of γ_*k*_, ϕ_*k*_, and *w*_*k*_(*s*(*i*), *s*_*k*_, **θ**) are used to propagate uncertainty through the prediction stage.

### Prediction stage

Results from the STEPPS pollen–vegetation calibration stage provide estimates of parameters that govern the pollen–vegetation relationship. Shifting from calibration to prediction requires assuming that the fundamental processes that link pollen and vegetation have remained unchanged over the last two millennia, as have the parameterizations of these processes. This assumption is standard in pollen–vegetation modeling and is a form of the broader assumptions of uniformitarianism in the geosciences: that the processes we observe today can be employed to gain insight into the unobservable and latent processes operating in the past. The key determinants of pollen dispersal and atmospheric entrainment, including size and shape of pollen grains and the morphology of male flowers and microstrobili, have not changed over the last two millennia (and beyond; Jackson and Lyford 1999). Canopy structure and roughness may affect pollen dispersal, but their effects are not well understood (Jackson and Lyford 1999). Pollen release from anemophilous trees generally occurs today under unstable atmospheric conditions (high turbulence; Jackson and Lyford 1999). Here we assume that this relationship of pollen entrainment and weather has remained unchanged in the past, an assumption of uniformitarianism.

Pollen productivity varies among individual trees, and also within trees over their life span (maturation, masting, interannual climate variation; Jackson 1994, Hicks 2001, Minckley and Shriver 2011). Although some determinants of productivity are conserved (i.e., pollen grains per anther or microsporangium, anthers per flower, microsporangia per microstrobilus), the number of flowers (or microstrobili) per tree or per unit biomass may be more variable. However, here we are primarily interested in relative pollen productivity, exploiting the fact that differences in pollen productivity are far greater among taxa than within taxa (Prentice 1988). For a more detailed discussions of these assumptions, we refer readers to Jackson and Lyford (1999) and Jackson (1994).

We emphasize that these assumptions are standard in palynology, and have been extensively and explicitly discussed and examined over the past several decades (Prentice 1988, Jackson 1994, Jackson and Lyford 1999). Framing a pollen–vegetation relationship in a Bayesian hierarchical context allows for the assessment of uncertainty in understanding of these processes during calibration, and establishes a natural framework for rigorous quantitative testing of hypotheses about temporally varying processes that affect pollen dispersal and production.

With the assumption that the distributions of these parameters remain constant throughout the considered time domain, information from the calibration stage can be used to estimate vegetation from pollen counts back through time.

The component of the prediction model linking pollen counts to inferred vegetation is identical to the calibration model, with added time dependence. Pollen counts at location *i* for time *t* for taxon *k* are denoted by *y*_*i*,*t*,*k*_ and are Dirichlet multinomially distributed according to

$$y_{i,t,\cdot }∼\text{DM}\left(n_{i,t},\varphi_{i,t}\right)$$

where $n_{i,t}=\sum_{k=1}^{K}y_{i,t,k}$ and the parameter $\varphi_{i,t,k}$ represents the sum of the local and nonlocal pollen contributions for taxon *k* such that

$$\varphi_{i,t,k}=\gamma \phi_{k}r_{s(i),t,k}+\frac{1}{C}\left(1-\gamma \right)\phi_{k}\sum_{s_{j}\neq s\left(i\right)}r_{s(i),t,}w\left(s\left(i\right),s_{j}\theta \right)\text{.}$$

In the calibration model, the proportional vegetation composition was known. In prediction, we seek to estimate proportional vegetation. The proportional vegetation for grid cell *s* at time *t* and for taxon *k* is denoted *r*_*s*,*t*,*k*_ and is linked to the corresponding taxon‐specific underlying spatial processes *g*_*s*,*t*,*k*_ through an additive log‐ratio sum‐to‐one constraint

$$r_{s,t,k}=\frac{\exp(g_{s,t,k})}{\sum_{k=1}^{K}\exp\left(g_{s,t,k}\right)}\text{.}$$

The underlying smooth spatial processes ***g***_*k*_ are normally distributed according to

$$g_{s,t,k}∼\text{Normal}\left(\mu_{s,t,k}^{g},\sigma_{s,t,k}^{2}\right),$$

where the process mean ($\mu_{s,t,k}^{g}$) is the sum of several terms. The process mean at the first time step is the sum of an overall adjustment term (μ_*k*_) and a spatial process term ($\nu_{s,k}^{s}$), both of which are time invariant. This time invariance of the process mean at *t* = 1 allows us to estimate the spatial processes at other times as deviations from the process at this initial time. For all other times such that *t* > 1, the process mean is the sum of the same overall adjustment and space‐varying term as before, but also a temporal (spatially invariant) term ($\mu_{t,k}^{t}$), and a term that accounts for the spatially correlated innovations that quantify the change across space between consecutive time steps ($\nu_{s,t,k}^{\mathrm{st}}$). More formally, we have that

(2) $$\mu_{s,t,k}^{g}=\left\{\begin{array}{cc} \mu_{k}+\nu_{s,k}^{s}, & \text{for}\;t=1\\ \mu_{k}+\nu_{s,k}^{s}+\mu_{t,k}^{t}+\nu_{s,t,k}^{\mathrm{st}}, & \text{for}\;2<t<T \end{array}\right.$$

The time‐varying term is given by the first‐order autoregressive model

(3) $$\mu_{t,k}^{t}∼\left\{\begin{array}{cc} \text{Normal}(0,\xi^{2}), & \text{for}\;t=2,\\ \text{Normal}(\mu_{t-1,k}^{t},\xi^{2}), & \text{for}\;3<t<T \end{array}\right.$$

Both the spatially varying mean and the spatial innovations must be estimated for each cell and for each taxon. Because of the complex dependence structure inherent in the model, the size of the domain, and computational limitations, we use the modified predictive process (Banerjee et al. 2008, Finley et al. 2009), which is a statistical representation of a spatial or spatiotemporal Gaussian process used to improve computational tractability. In brief, in the modified predictive process, the spatial process is estimated at a number of spatial locations referred to as knots s* in the spatial domain, where the number of knots is fewer than the original number of spatial points in the domain (Finley et al. 2009). A transformation and variance correction are then applied to the knot location process estimates to obtain estimates for the original domain.

According to the modified predictive process, the spatial‐varying time invariant term can be represented as

$$\nu_{\cdot ,k}^{s}=c_{k}\left(s,s^{*};\eta_{k},\rho_{k}\right)C_{k}^{*-1}\left(s^{*};\eta_{k},\rho_{k}\right)\alpha_{\cdot ,k}^{s},$$

where

(4) $$\alpha_{\cdot ,k}^{s}∼\text{MultivariateNormal}\left(0,C_{k}\left(s^{*};\eta_{k},\rho_{k}\right)\right)$$

and

(5) $$C_{k}\left(s^{*};\eta_{k},\rho_{k}\right)=\eta_{k}^{2}\exp\left(-d\left(s^{*}\right)/\rho_{k}\right),$$

(6) $$c_{k}\left(s,s^{*};\eta_{k},\rho_{k}\right)=\eta_{k}^{2}\exp\left(-d\left(s,s^{*}\right)/\rho_{k}\right)\text{.}$$

*C*_*k*_ and *c*_*k*_ are covariance matrices that quantify the spatial covariance between pairs of knot locations and pairs of knot and cell locations, respectively. The covariance is modeled using an isotropic, exponential covariance that decays as a function of distance. Covariance depends on the absolute distance between pairs of locations, denoted by the distance matrices *d*(s*) and *d*(***s***,s*) with dimensions *N*_knots_ × *N*_knots_ and *N* × *N*_knots_. Covariance parameters η_*k*_ and ρ_*k*_ determine the amount of spatial smoothing, and are estimated a priori by fitting a modified predictive process model with identical knots and spatial domain to the settlement‐era composition data.

The innovations that result from differences in consecutive time steps also follow a modified predictive process, where

$$\nu_{\cdot ,t,k}^{\mathrm{st}}=c_{k}\left(s,s^{*};\eta_{k},\rho_{k}\right)C_{k}^{*-1}\left(s^{*};\eta_{k},\rho_{k}\right)\alpha_{\cdot ,t,k}^{t},$$

and are autoregressive where

$$\begin{array}{cc} \alpha_{\cdot ,t,k}^{t}∼ & \text{MultivariateNormal}(\omega \cdot \alpha_{\cdot ,t-1,k}^{t},\left(1-\omega^{2}\right)\cdot \\ & C^{*-1}\left(s^{*};\eta^{2},\rho_{k}\right))\text{.} \end{array}$$

The parameter ω defines the behavior of the innovations and specifies constant prior variance across time. Using an autoregressive component allows the data to borrow strength across time but relies on the assumption composition changes smoothly through time. This representation may smooth over abrupt changes.

The process variance term $\sigma_{s,t,k}^{2}$ is the sum of three terms; two are variance corrections associated with the predictive processes that represent the spatially varying mean and the autoregressive innovations; these corrections capture the additional fine‐spatial‐scale variability that cannot be represented by the predictive process approximation. The third term is commonly referred to as a nugget, which describes the variance at a single point in time and space due to fine‐scale variability not accounted for in the model. Then we have that

$$\begin{array}{cc} \sigma_{s,t,k}^{2}=\; & \eta_{k}^{2}-\eta_{k}^{2}{\left(c\left(s,\cdot \right)C^{*-1}c{\left(s,\cdot \right)}^{\prime }\right)}_{s}+\tau^{2},t=1\\ \sigma_{s,t,k}^{2}=\; & \eta_{k}^{2}-\eta_{k}^{2}{\left(c\left(s,\cdot \right)C^{*-1}c{\left(s,\cdot \right)}^{\prime }\right)}_{s}+\left(1-\omega^{2}\right)\cdot \\ & \eta_{k}^{2}-\left(1-\omega^{2}\right)\cdot \eta_{k}^{2}{\left(c\left(s,\cdot \right)C^{*-1}c{\left(s,\cdot \right)}^{\prime }\right)}_{s}\\ & +\tau^{2},t>1 \end{array}$$

In the case where a knot and cell have equal coordinates, the distance between them will be zero, resulting in a cross‐covariance of one and a variance correction equal to η^2^. When this correction is subtracted from the variance of the parent process we obtain a difference of zero. This is not necessarily cause for concern—the estimated process at that cell is equal to estimated process at the knot. However, to simplify computational implementation we define a nonzero nugget τ^2^ = 1 × 10^−5^ that is added to the process variance at each cell to ensure nonzero variance.

### Spatial and temporal resolution of predictions

The prediction model estimates composition for discrete points in space and time. The spatial resolution of predictions is constrained by the resolution of the gridded PLS data, the ability of dispersed networks of pollen sites to resolve fine‐scale heterogeneity in vegetation composition, and computational limitations. For these reasons, composition predictions are made on a regular square grid with cells that are 24 × 24 km.

To account for pollen sample age uncertainty, 40 sets of age posterior draws are randomly selected. For a given site, each posterior draw represents a distinct parameterization of the age‐depth model. For each of these 40 sets of age posteriors, and for each site, pollen samples are binned in 100‐yr intervals from 2,150 to 150 YB1950. STEPPS is then used to infer vegetation composition for each of these 40 pollen data sets.

The discrete centennial‐scale time discretization is needed for computational tractability and increases the effective sample size for each time interval, but decreases temporal precision. Additionally, we note that because of the spatiotemporal autocorrelation in the model, subsequent temporal estimates are not truly independent; changes that the model detects emerge despite the modeled dependence. Pollen samples that represent postsettlement forests are excluded because of this paper's focus on presettlement vegetation change. Samples were classified as postsettlement if they were younger than either the expert‐identified Euro‐American settlement horizon (Dawson et al. 2016) or 150 YB1950 (based on age‐depth models).

### Computation

Parameters were estimated using the adaptive No‐U‐Turn Sampler (NUTS), a variant of the Hamiltonian Monte Carlo method (Hoffman and Gelman 2011). The dependence structure of the model in both time and space results in a computationally intensive analytic expression for the mathematical gradient of the joint log posterior. The complexity of the model gradient prevented the use of automatic differentiation as implemented in the Stan software (Stan Development Team 2017). To overcome this challenge, we used the Stan implementation of adaptive NUTS (Stan v2.17.1) with a hard‐coded implementation of the mathematical gradient. Additionally, we used openMP to parallelize the evaluation of gradient and joint posterior functions (Dagum and Menon 1998). All postprocessing and analysis of posterior parameters estimates was completed in R (version 3.4.2; R Development Core Team 2013).

### Analysis of forest composition estimates

The prediction model estimates spatial patterns of forest composition over time. In this work our interest lies in regional‐scale changes estimated by the Gaussian process mean $\mu_{s,t,k}^{g}$ (recall that *s* refers to the grid cell, *t* refers to the time, *k* refers to the taxon, and the superscript denotes that this mean is associated with the gaussian process denoted by *g*). This process mean is the smooth portion of the spatial variation in the proportional presence of a given taxon, namely, the portion that can be represented by the predictive processes. The full spatial process, *g*_*s*,*t*,*k*_, includes the mean $\mu_{s,t,k}^{g}$, the variance corrections for each of the predictive process components, and an additional (but negligible) nugget effect (described in the *Prediction stage* section). This additional heterogeneity from the variance corrections allows the model to represent the variability in the data better; however, the process mean better represents the smooth large‐scale patterns.

To determine statistical significance, for each posterior draw (which consists of estimates of the proportional abundances for many individual tree taxa through space and time) we calculated the difference in proportional abundance for a given taxon between all pairwise combinations of times for each grid cell in the spatial domain. For a given grid cell and comparative pair of time periods, we determined the probability of a directional change in proportional abundance. Changes with a probability greater than or equal to 0.85 are considered statistically significant; we note that in a Bayesian framework, this is analogous to the statement that significant changes are those that occurred with 85% certainty. Note that this cutoff is relaxed slightly from standard thresholds (e.g., 0.95) to accommodate the noisiness of pollen data relative to the trends of the last two millennia.

An artifact of the statistical model is that it predicts tree proportions to be nonzero throughout the entire spatial domain for all taxa. However, for certain taxa we know that their ranges do not cover the entire spatial domain. For any statistically significant changes to be deemed ecologically significant, we required that the relative composition of the taxon be greater than 3% for at least one of the pair of times being compared; this cutoff was based on expert elicitation about the possibility of using pollen records to identify changes in taxa that are low in relative abundance. We note that this cutoff is conservative; a lower cutoff would have resulted in the claim that more statistically significant changes occurred. However, when relative abundances get well below 3%, it becomes increasingly difficult to tease apart statistical artifacts from true signal. This is not to say that uncommon taxa lack ecological importance, but uncertainty roughly scales according to the range of observed percentages. As such, for these less common taxa there is lower certainty relative to the variation.

The tests described allow us to identify statistically and ecologically significant change. However, the posterior distributions provided by STEPPS enable more detailed inference about change in relative abundance. Specifically, we can test for both statistically significant large changes and statistically significant stability. When comparing large relative abundance estimates for pairs of time intervals for each grid cell, we can distinguish among three cases: (1) a taxon experienced large and significant changes in relative abundance; (2) a taxon was significantly stable; and (3) neither of the previous two cases applies. To do this we test if the differences in relative composition for a pair of time points and a spatial location are significantly above 5% (large change threshold) or significantly below 3% (stability threshold). These thresholds are subjective but well aligned with prior ecological interpretations of fossil pollen records, which commonly use thresholds of 0.5% to 5% for visualization and analysis of pollen data as rough indices of species presence (Van der Knaap et al. 2005). In subsequent analyses, we identify locations with at least one taxon experiencing large change and locations of significant stability across all taxa (where every taxon is significantly stable).

Confidence in identifying changes in the abundance for each taxon in any given grid cell is a function of the posterior distribution of the estimated relative abundance of trees in that grid cell. The shape and spread of that distribution is a combined function of the uncertainties estimate by STEPPS, including the calibrated pollen–vegetation relationship for each taxon, dating uncertainty, and the consistency in signal across space and time. To illustrate how some of these factors influence statistical certainty about composition change, we chose two example taxa: beech and pine (Fig. 2). Beech is found only in a subset of our spatial domain, but the proportion of beech pollen has a relatively robust relationship with the proportion of beech trees on the landscape (Webb et al. 1981, Jackson and Kearsley 1998, Paciorek and McLachlan 2009, Dawson et al. 2016). Pine is distributed across the UMW domain, and is known to have overdispersed pollen. To examine the relationship between changes in relative abundance and uncertainty of these changes, we compute and map the mean and standard deviation (from the posterior distribution) of differences in relative abundance for pairs of time points for each grid cell.

### Ecotone analysis

We represent the position and shifts in ecotones via empirical orthogonal function (EOF; see Cressie and Wikle 2015) analysis to decompose the high‐dimensional forest composition data into dominant patterns of variability. EOF analysis was completed using singular value decomposition (Cressie and Wikle 2015). The spatial pattern of the first principal component of the EOF analysis closely corresponds to the long‐recognized tension zone between northern mixed forests and the temperate hardwoods and oak savannas to the south (Curtis 1959).

For visualization purposes, we delineate the tension zone ecotone as a line that represents the point at which the first principal component changes sign (i.e., where the first EOF is zero). This line represents the most abrupt transition between the northern and southern forest communities. However, we keep in mind that the transition between these forest communities may occur abruptly or gradually; in both cases it is possible to demark the position of greatest community turnover. We then represent shifts in the position of the ecotone by creating a transect that is roughly perpendicular to the ecotone and quantify the movement of the ecotone line as kilometers of movement along each of these transects, relative to the ecotone position at the most recent time interval used in analysis (350–250 YB1950).

## Results

Forty prediction model runs were completed, with a warm‐up period of 250 iterations and a sampling period of 2,000 iterations. For each run, pollen sample ages and calibration parameters were drawn from previously generated posterior distributions for these quantities (Dawson et al. 2016) and provided as model input. Initial conditions varied among runs, and were determined using the default methods implemented in Stan (Stan Development Team 2017). Posterior parameter estimates were combined across runs, and every 40th sample was retained for further processing (for computational tractability). Initial assessment of the results suggested that agroindustrial signals were present at 200 YB1950; as a result, we excluded this time interval from all further analysis to avoid erroneously reporting agroindustrial impacts as natural variability. Code used to estimate model parameters and perform the analysis, and posterior draws for the relative abundance vegetation predictions, are archived and available; see the *Data Availability* statement.

### Community compositional change

Higher pollen site density tends to improve confidence in detecting compositional change (Fig. 2). However, increasing site density does not necessarily reduce uncertainty. The relationship between sediment pollen and vegetation is a noisy process, with uncertainty arising from measurement, radiometric dating, age‐depth modeling, dispersal, deposition, sedimentation, and other sources. For beech, change greater than 10% in relative abundance seems to signify statistically robust vegetational change (Fig. 2, upper panels). However, for pine, even changes in mean magnitude as large as 15% are hard to interpret with confidence (Fig. 2, lower panels). This may in part be because there are three species of pine in the region, but only a single beech species, or because pine pollen is more subject to interannual fluctuations in productivity and atmospheric transport.

**Figure 3:**
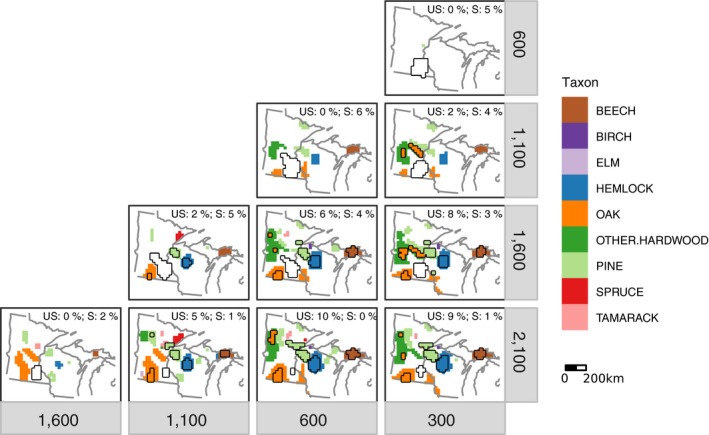
For pairs of time intervals in YB1950, maps indicate regions of (1) large change (increase or decrease) and (2) significant stability. Colors depict the taxon that experienced the greatest large change in abundance in any given grid cell, where large change is defined to be greater than 5% change in abundance. Regions of statistically significant large change (i.e., those with at least one taxon that changed by at least 5% with posterior probability of change greater than 85%) are outlined in black. White regions outlined in black indicate significant stability (i.e., there is a probability greater than 85% that all 12 tree taxa experienced less than 3% change between a pair of time intervals). Regions that are white and not outlined indicate locations where no taxon changed by more than 5% and are not significantly stable. Text in the upper‐right corner indicates the percent of cells (relative to total number of cells in the domain) that (1) experienced large significant change (US for unstable), and (2) were significantly stable (S for stable). Note that it is easiest to read the figures comparing pairs of time intervals diagonally from lower left to upper right.

At the level of communities, and across the UMW in general, most locations did not experience large change over the 2,100–300 YB1950 time period considered (Fig. 3). Similarly, most locations were not significantly stable. Instead, most locations experienced either (1) small change, defined to be less than 5% but statistically different from zero; or (2) uncertain change, defined to be change not statistically different from zero. This suggests that there is not sufficient evidence to refute the Frelich statement about vegetation stability. Over the longest period preceding the impact of Euro‐American influence (2,100–300 YB1950), approximately 9% of grid cells in the UMW experienced significant composition change. Areas of significant change are localized to hotspots in north‐central Wisconsin, a strip in central Minnesota, western Wisconsin, and the Upper Peninsula of Michigan (Fig. 3, all panels).

**Figure 4:**
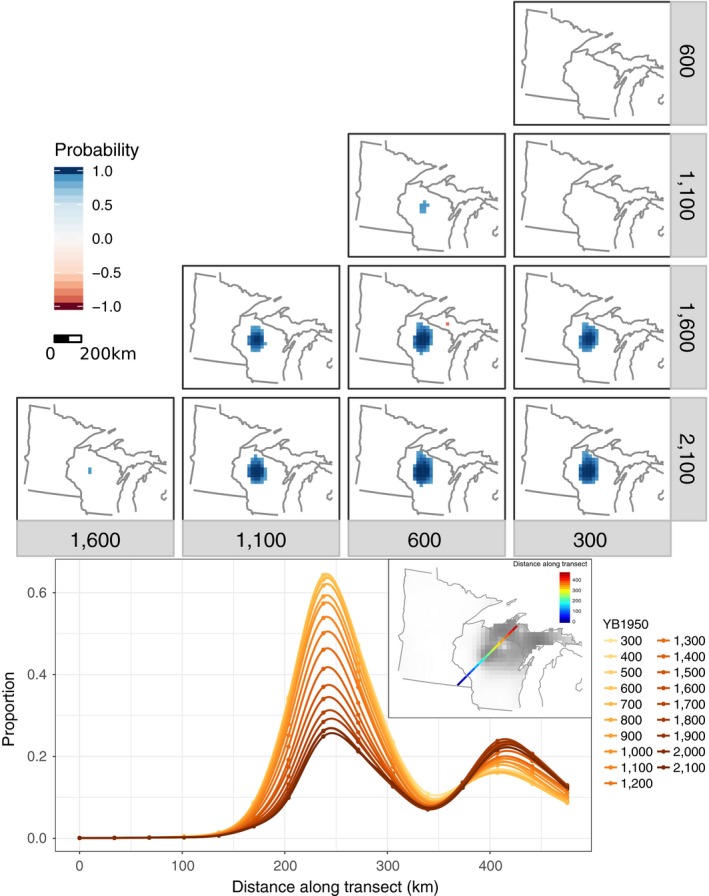
Upper panel: Regions of significant change in hemlock between pairs of time intervals (only posterior probabilities greater than 85% are plotted). Blue indicates an increase in the more recent time, and red indicates a decrease. Lower panel: Proportion of hemlock composition along the transect indicated in the subset panel, at century intervals.

**Figure 5:**
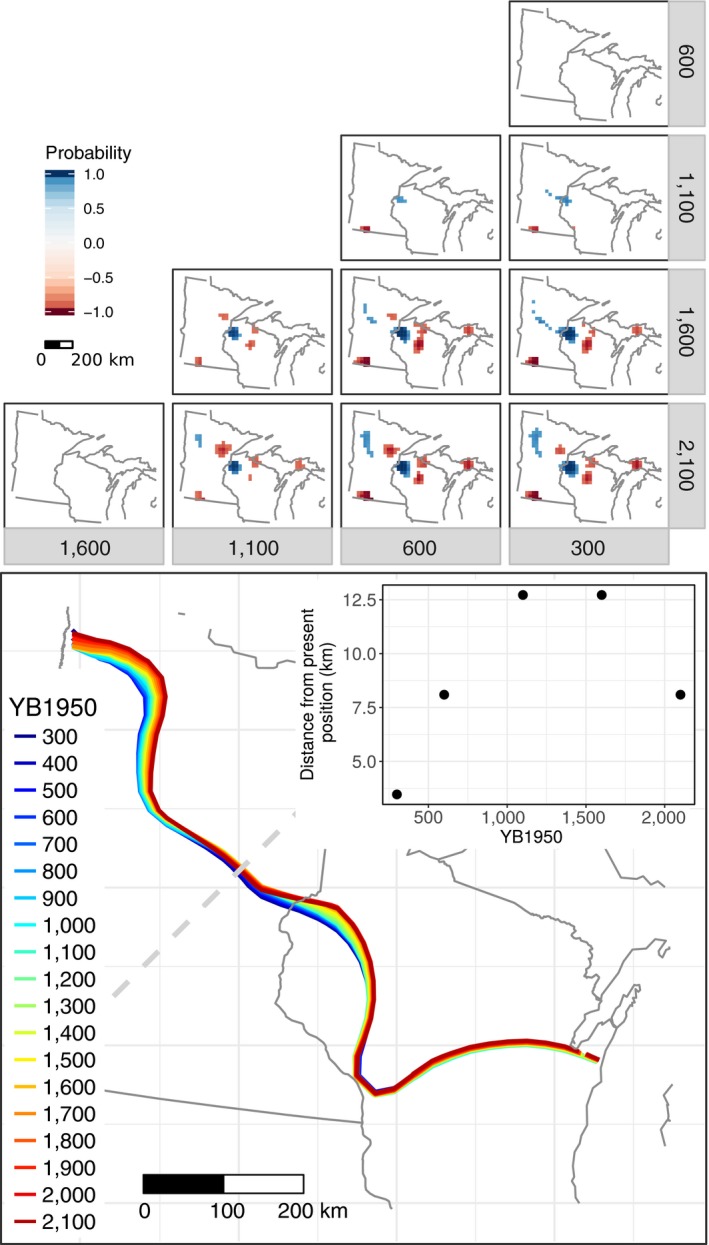
Upper panel: Regions of significant change in pine between time intervals. Blue indicates an increase in the more recent time, and red indicates a decrease. Lower panel: The position of an ecotone for each century, identified by EOF analysis roughly corresponds to the Tension Zone (Curtis 1959). Subset figure shows the ecotone movement in kilometers from the 300 YB1950 position, obtained by comparing ecotone position for consecutive time intervals 200 yr apart, along the indicated transect.

**Figure 6:**
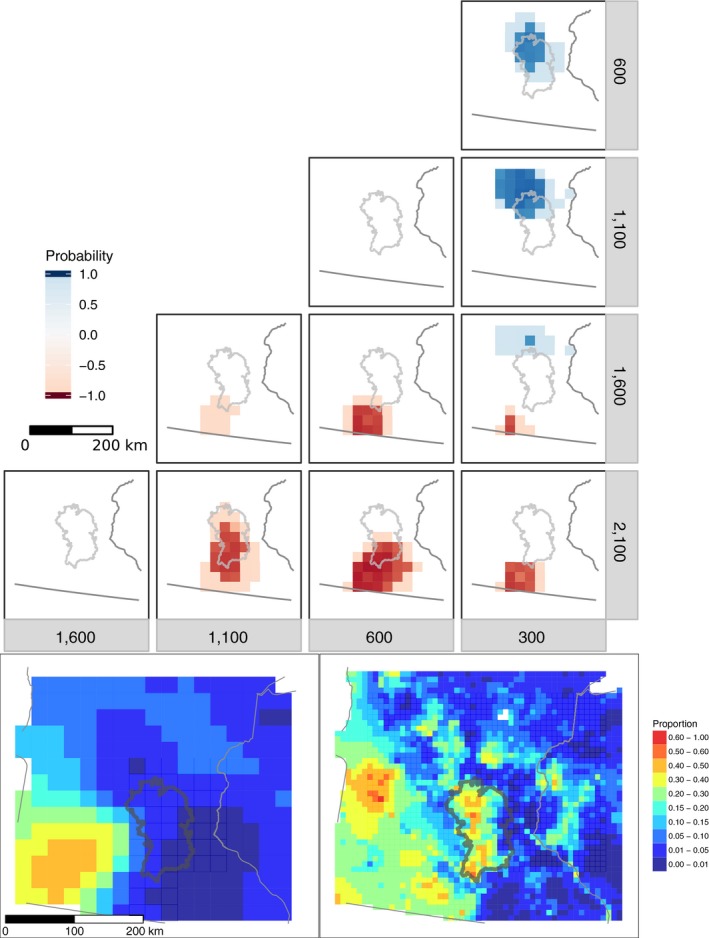
Upper panel: Regions of significant change for the sum of ash and elm, two key Big Woods taxa, between time intervals. Blue indicates an increase in the more recent time, and red indicates a decrease. Lower panel: Composition for the sum of ash plus elm as predicted by STEPPS at 300 YB1950 (left) and in the Public Land Survey data (right). Gray boundary indicates the Big Woods Ecological Subsection defined by the Minnesota Department of Natural Resources.

Each of these hotspots represents a different combination of tree species. The changes in central Wisconsin and the upper peninsula of Michigan can be attributed to rising proportions of hemlock and beech over the last two millennia (Fig. 4), whereas the band of community‐level change in central Minnesota and western Wisconsin can be attributed to increases in pine and a small southward shift of the ecotone between northern mixed forests and southern hardwoods (Fig. 5). At the same time, community‐level analyses can obscure ecologically (and statistically) significant changes at the taxon level, particularly those for less common taxa, such as the rise of mesic hardwoods and establishment of the Big Woods in southern Minnesota (Fig. 6). We review taxon‐level dynamics more fully in the following sections.

These changes in community composition accumulated over multiple centuries. Pairwise comparisons of maps spaced a few centuries apart (main diagonal in Fig. 3) generally do not show significant change, except between 300 and 200 YB1950, in the earliest era of Euro‐American land use (not shown).

Across the entire region, the largest proportion of area to experience significant landscape change was between 1,600 and 1,100 YB1950 Fig. 3), driven primarily by increases in hemlock (Fig. 4) and pine (Fig. 5).

### Hemlock

Of all individual taxa, hemlock had the most grid cells with significant changes prior to 1,200 YB1950. At the settlement era, its contiguous range extended from the Upper Peninsula of Michigan to central Wisconsin (Davis et al. 1986) although it was patchily distributed to southwest Wisconsin and the eastern edge of Minnesota (Calcote 1987). Changes in hemlock abundances over the last 2,000 yr have been episodic, with little apparent change in overall range extent but significant within‐range changes in relative proportion (Fig. 4). The relative abundance of hemlock increased in the western portion of hemlock's range in central Wisconsin from about 2,100 to 600 YB1950, with the most accelerated increase from 1,600 to 1,100 YB1950. Conversely, hemlock abundances significantly declined in the Upper Peninsula of Michigan between 1,600 and 600 YB1950. Beginning at 300 YB1950 hemlock abundance declined throughout a large portion of its range in the UMW (not shown). This decline may have been linked to changes in land‐use patterns (Muzika et al. 2015); a more thorough analysis of land‐use histories in the region is needed to support this hypothesis.

### Beech

Significant and large increases in beech occurred in the Upper Peninsula of Michigan during the last 2,000 yr. These increases appear to have commenced around 2,100 YB1950, continuing until ca. 600 YB1950 (Fig. 3). In the center of its range in the Upper Peninsula, the relative abundance of beech increased from 20% to more than 50% within this time period. To the east, in Lower Michigan and adjacent Ontario, beech populations decreased between 1,000 and 600 YB1950 (Booth et al. 2012b). Beech decreased throughout its range in our study area between 600 and 300 YB1950. However, these decreases were minor; relative abundance of beech after this decline remained higher than beech abundance 2,100 YB1950. Similar to hemlock, the late Holocene beech increase was unaccompanied by an expansion in geographic range.

### Northern mixed/southern hardwoods ecotone

The position and dynamics of the ecotone between northern mixed forests and southern hardwood forests is well constrained by existing paleodata networks (Fig. 1), particularly in Minnesota, and offers an example of an ecological phenomenon that was largely stable over the last 2,000 yr, albeit with some movement (Fig. 4). The ecotone appears to have shifted southwestward by about 8–30 km (Fig. 5, lower) over the last 2,000 YB1950, with the largest shifts in northwestern Minnesota and southward shifts throughout Minnesota and northwestern Wisconsin. In central Wisconsin, there is no detectable movement of the ecotone.

The shifts in the ecotone can be attributed to the southwestward expansion of pine populations (Fig. 5, upper, and Fig. 3). In Minnesota, the ecotone is characterized in part by a transition from pine‐ or tamarack‐dominated forests in the northeast to oak‐dominated forests in the southwest (Appendix S1: Fig. S1), with the ecotone situated close to the point at which the relative abundance of pine approximately equals that of oak.

These findings agree qualitatively with prior reports of increases in *Pinus strobus* in northwestern Wisconsin (Tweiten et al. 2015) and both *P. strobus* and *Pinus banksiana*/*resinosa* in north‐central Minnesota (McAndrews 1967, Jacobson 1979) over the last 2,000 YB1950. Earlier ecological interpretations of palynological time series, however, were confounded by the challenge of disentangling local from extraregional sources of *Pinus* pollen (McAndrews 1967), so these modeling efforts add value both by identifying specific regional loci of changing *Pinus* abundances (Fig. 5, upper) and by adding quantitative rigor to the long‐established efforts to infer spatial shifts in emergent ecological phenomena (position of vegetation formations, shifts in ecotones, etc.) from networks of individual, site‐level palynological records (Webb et al. 1983, Williams et al. 2009).

### Big Woods expansion

Ash and elm are characteristic of the mesic deciduous Big Woods and are used here as indicators of the late‐Holocene formation of the Big Woods (Grimm 1983). The rises in ash and elm abundances between 600 and 300 YB1950 are significant and indicate that the formation of the southern Minnesota Big Woods is relatively recent (Fig. 6). This result agrees with regional reconstructions of vegetation history based upon qualitative interpretations of fossil pollen records (McAndrews 1968, Grimm 1983, Umbanhowar et al. 2006), but, for the first time, the magnitude of the rise can be quantified (the relative abundance of ash and elm trees increased from approximately 2–5% on average over this time period) and its statistical significance demonstrated.

The areas of statistically significant changes in the sum of ash and elm are closely centered over the Big Woods region (Fig. 6, upper). One caveat is that the Big Woods region at the presettlement era is only weakly expressed in maps of STEPPS‐estimated ash and elm for 300 YB1950, despite being apparent in maps of the PLS data (Fig. 6, lower). The same is true for the predictions at 200 YB1950 (not shown). This discrepancy may be attributed to the inconsistent pollen signal for elm in that region; some pollen records from the Big Woods region indicate that elm increases to about 10% relative pollen abundance, whereas other records from that region do not (Umbanhowar 2004). However, it is also possible that the hallmark Big Woods taxa increased in relative abundance after the time period considered here. In this paper, the most recent predictions were for 200 YB1950; however, most PLS survey dates fall in the second half of the 19th century—approximately 100 yr after the our most recent predictions.

## Discussion

### Overview

An advantage of integrated statistical analyses, like STEPPS, is the ability to borrow strength in the data across both space and time, producing estimates of composition smoothed across tens of kilometers and over a few centuries, with quantification of the associated uncertainty. Here, we quantified changes in forest composition in a way that accounts for the major uncertainties in paleoecological data. Although the application here is to the upper Midwest, the STEPPS modeling framework is general and can be applied to other regions that have spatial networks of pollen and vegetation data (for calibration) and fossil pollen records (for prediction). The diverse set of dynamics documented here, varying across space and across taxa, provides a rich ground for further understanding the processes governing forest dynamics across a range of timescales. Here we first review the patterns and insights gained into forest dynamics in the UMW over the last two millennia, then discuss the calibration and smoothing associated with STEPPS and the advantages of using interated statistical models like STEPPS for inference.

### Vegetation dynamics in the upper Midwestern US: a landscape of change and relative stability

Vegetation dynamics in the UMW over the last two millennia were complex, so a key advantage of the STEPPS approach is its ability to quantify and delineate this complexity. Most places in the UMW experienced either small or uncertain change in forest composition during the 2,000 yr before Euro‐American settlement, and a few places and taxa changed substantially. Some taxa experienced large changes (i.e., absolute change >5% in relative abundance with probability >0.85) in abundance, as did community composition in some places (i.e., absolute change >0% in relative abundance of with a probability >0.85). These changes tended to be localized and within a regional background of compositional change that was on average small in magnitude and often not significantly different from zero. However, community stability without significant change in any species is strongly statistically supported only in southeastern Minnesota.

The most dramatic change in the preindustrial forest was the explosion of hemlock abundance in central Wisconsin (Fig. 4), accompanied by a rise in beech in northern Michigan. The Midwestern expansion of hemlock has been studied intensively (Davis et al. 1986, Davis and Sugita 1997), so the main contributions here are: (1) the formal quantification of magnitude and significance, and (2) establishing the anomaly of the large hemlock rise relative to the more muted changes for other taxa. At the epicenter of the hemlock expansion, hemlock abundance doubled, going from 20% to 40% in the first thousand years of our analysis, followed by a further 20% increase in the next thousand years. Remarkably, although this expansion took place within 100 km of the species’ western range limit, the increase in abundance shows no signal of an accompanying westward expansion of hemlock's range, although this analysis may miss the establishment of small outlier populations (Parshall 2002). Similarly, hemlock abundances 100 km east of the hemlock growth peak were stable (Fig. 4). This rapid but localized population growth rate of a species is consistent with forest simulations of hemlock dynamics, given its restricted seed dispersal and slow but persistent growth in low light (Pacala et al. 1996).

The local expansion of hemlock in north central Wisconsin was synchronous with other shifts in tree taxa, perhaps suggesting a common forcing. To the west of the peak of hemlock, pines increased in abundance and, to the east, beech populations expanded (Fig. 3). As for hemlock, the beech expansion was local, with no signal of population growth elsewhere in the study area (Appendix S1: Fig. S3b). In fact, beech populations underwent a decline further east, in the central Great Lakes region between 1,000 and 600 YB1950 (Booth et al. 2012b).

A key feature of STEPPS is that its posterior taxon‐level estimates can be readily analyzed to quantify and assess significance of community‐scale features such as ecotone shifts. We found that, over the last 2,000 yr, the expansion of northern forest taxa into oak forests was highest in northwest Minnesota (40 ± 5 km), but we detect almost no movement of the southern boundary in central Wisconsin (but note the sparsity of pollen records in that area: Fig. 1). As with hemlock, our work provides quantitative backing for previous studies that have identified both the general shift toward more northern forest types along this ecotone and heterogeneity in the rates of this shift (McAndrews 1966, Jacobson 1979, Almendinger 1992, Nelson and Hu 2008, Williams et al. 2009), but were not able to formally assess the significance of these changes.

For most locations and times we find no statistically significant evidence for either large change or stability. The expected change in any taxon over any 500‐yr interval is less than 2%. For any 1,000‐yr interval, the change in posterior mean for any taxon is less than 3%. Even across the largest time interval of almost 2,000 yr, only 9% of grid cells showed significant changes in any taxon greater than 5% (Fig. 3). The aggregate data across the region hence do not suggest large shifts in vegetation over most areas over the 2,000 yr before Euro‐American settlement. Hence, for many regions of the UMW, assumptions of late Holocene stability prior to Euro‐American settlement (Frelich 1995, Schimel et al. 2013), or at least changes too subtle to be detected confidently, are supported by the STEPPS model.

Although we do not formally assess here the role of potential environmental drivers on these vegetation changes, many of the above changes are consistent with regional trends toward cooler and moister conditions over the last 2,000 yr (Marlon et al. 2017). There is also strong evidence that extensive droughts (Booth et al. 2012b) and changing fire regimes (Umbanhowar 2004, Tweiten et al. 2015) have affected UMW vegetation. A next key step is to combine these vegetation reconstructions with climate reconstructions for the last several millennia (Ahmed et al. 2013) to understand better the combination of external forcings, internal feedbacks, and interaction of slow and fast processes (Svenning and Sandel 2013) that produced the observed mixture of forest change and stability.

### STEPPS calibration, smoothing, and signal‐to‐noise

STEPPS is well calibrated across our entire study area (Dawson et al. 2016), so estimates of taxon abundance across our study area should be unbiased. PLS data are affected by surveyor biases (Kronenfeld and Wang 2007, Bouldin 2008), but these effects are strongest for estimates of stem density and biomass, which are not used here, and the PLS‐based data layers used here include corrections for these biases (Goring et al. 2016, Cogbill et al. 2018).

The STEPPS model as implemented here is best suited for detecting multi‐centennial‐scale changes in forest composition. By borrowing strength across space and time, the model induces some spatiotemporal smoothing in estimates of compositional change, so rapid or local changes in pollen abundances might be smoothed. The Big Woods provide an example of how STEPPS interprets rapid local changes. The expansion of Big Woods mesic hardwood forests over the last millennium is well established (Grimm 1983, Camill et al. 2003), but STEPPS is confident that all changes in abundance were less than 3% (Fig. 3). In the Big Woods region (Fig. 6), the combination of noisiness in pollen data and the coarse resolution of the 24‐km grid tends to smooth out local variation in vegetation. STEPPS should accurately capture the magnitude of regional changes across grid cells at time intervals longer than a few centuries.

Analyses of these posterior estimates show how estimating vegetation changes from fossil pollen networks can be affected by variations in signal‐to‐noise ratios within and across taxa (Fig. 2). These ratios can change spatially as a function of site density and abundances of other taxa. STEPPS usually can confidently identify a 10% change in hemlock abundance, but a 10% change in pine is only identifiable in some locations, because the taxa have different signal‐to‐noise ratios (Fig. 2). Increasing site density does not always help reduce these uncertainties, because of inherent noise in the process of translating pollen abundance to tree abundance. Such issues have been well known in paleoecology since its inception (Von Post 1916, Edwards et al. 2017), but full representation of these uncertainties has been difficult for traditional statistical methods.

### Advantages of STEPPS modeling framework

An integrated statistical approach to ecological inference from paloecological data carries several advantages. First, it allows an even‐handed analysis of spatiotemporal trends. STEPPS is well calibrated against the UMW settlement‐era pollen and vegetation data sets (Dawson et al. 2016), so estimates of taxon abundance and uncertainty scale appropriately over space and time. Uncertainty is accordingly amplified in locations far from fossil sites. At the resolution allowed by the data, we can determine which time periods and spatial patterns emerge beyond the noise, and that determination is consistent with prior knowledge. Maple, for instance, is known to be poorly represented in sedimentary pollen (Jackson 1994), and our reconstruction of maple abundance is appropriately uncertain (Appendix S1: Fig. S3f, S5f). Accounting for the multiple sources of uncertainty in paleodata helps gain confidence that significant changes, when identified, represent real ecological signals (e.g., Fig. 3).

Second, calibration against forest data allows us to make statistical inference about the long‐term population and community trends of trees, rather than of raw pollen abundances, in terms and units relevant to contemporary forest ecologists. The doubling time of hemlock in central Wisconsin, for instance, was approximately 600 ± 200 yr, and we can say with some degree of statistical confidence that this increase was not accompanied by detectable westward expansion (Fig. 4). Understanding the long‐term population growth rates of trees (Giesecke et al. 2017) and the rates of biome shifts (Williams et al. 2004) are of immense practical importance (Iverson et al. 2004), but palynology has always faced the challenge of discriminating palynological and taphonomic artifacts from ecological signals (Jackson 1994).

Third, because of this challenge, sometimes paleoecologists may have erred on the side of caution, underemphasizing trends that were apparent in the data but were difficult to report without a formal uncertainty analysis. Hence, this statistical approach allows identification of previously unidentified features of the preagroindustrial landscape. For example, the spread of hemlock toward its western range boundary has been widely reported (Davis et al. 1986), but changes in population size have not been statistically estimated. In prior papers (Heide 1984), the rapid expansion of hemlock is apparent in the data, but was not commented upon. By borrowing strength across all pollen counts in our region, STEPPS was able to identify previously unreported trends and better quantify previously observed trends.

Finally, STEPPS’ process‐based modeling of vegetation–pollen relationships and associated uncertainties create new opportunities to use the recent fossil record to constrain ecological forecasts for coming decades. STEPPS is one of an emerging class of modeling frameworks known as proxy system models (Evans et al. 2013), which aspire to represent the processes by which environmental and ecological processes are recorded in proxy observations. The posterior estimates from STEPPS, which embed and integrate multiple sources of uncertainty, can be used via state‐ or parameter‐data assimilation to test and refine the predictive ability of forest simulators, helping close the loop between observational data and mechanistic models (Dietze et al. 2018), at timescales beyond that available to the instrumental record.

## Data Availability

Code used to estimate model parameters and perform the analysis is available through https://doi.org/10.5281/zenodo.3350906 Posterior draws for the relative abundance vegetation predictions are archived and available through https://doi.org/10.6073/pasta/c941fc6bd9eb959384592766dc0e9dbb
