# Supplementary material for: Quantifying trends and uncertainty in prehistoric forest composition in the upper Midwestern United States
Source: Ecology. 2019 Sep 13;100(12):e02856. doi: 10.1002/ecy.2856 (PMC6916576; doi:10.1002/ecy.2856)
Supplement: Supplementary file 1 [file ECY-100-na-s001.pdf]

Supporting Information. Dawson, Andria, Christopher J. Paciorek, Simon Goring, Stephen T. Jackson, Jason A. McLachlan, and John W. Williams. 2019. Quantifying trends and uncertainty in prehistoric forest composition in the upper Midwestern United States. *Ecology*.

Appendix S1.

All time units for figures in this appendix are in units of YB1950.

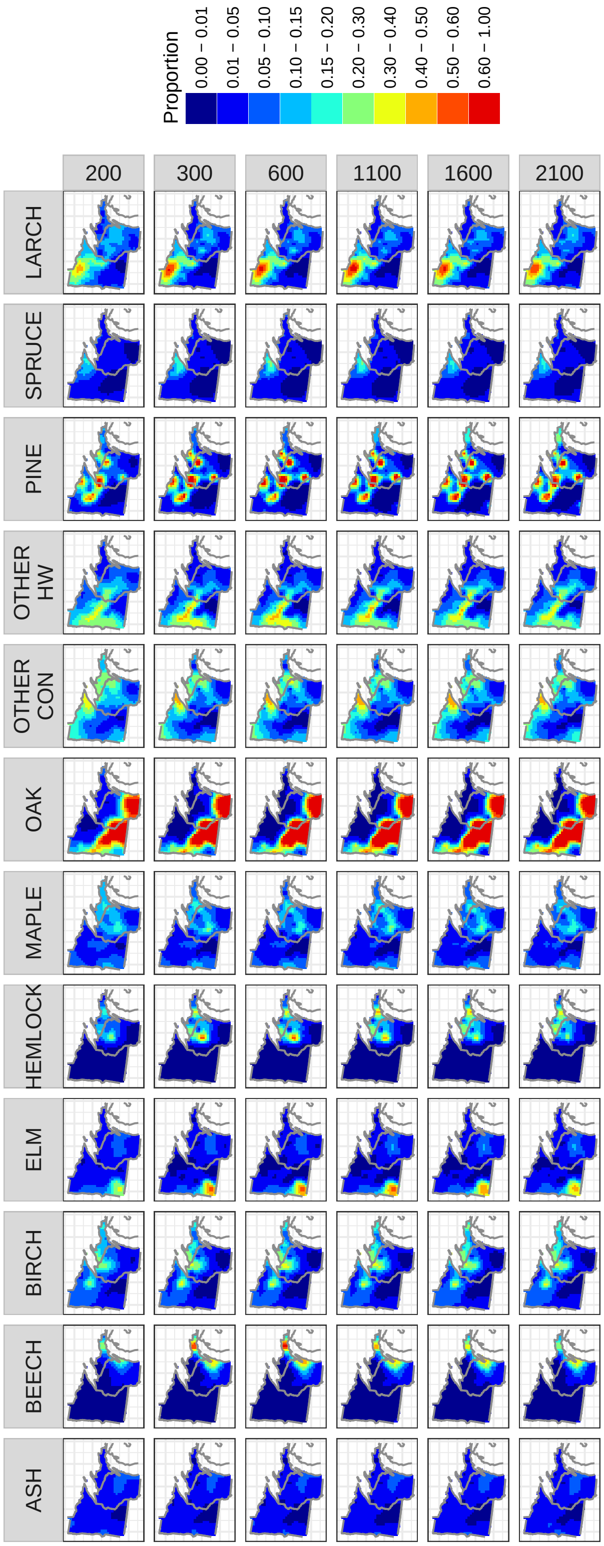

Figure S1: Mean estimates of relative composition by taxon for a subset of time intervals.

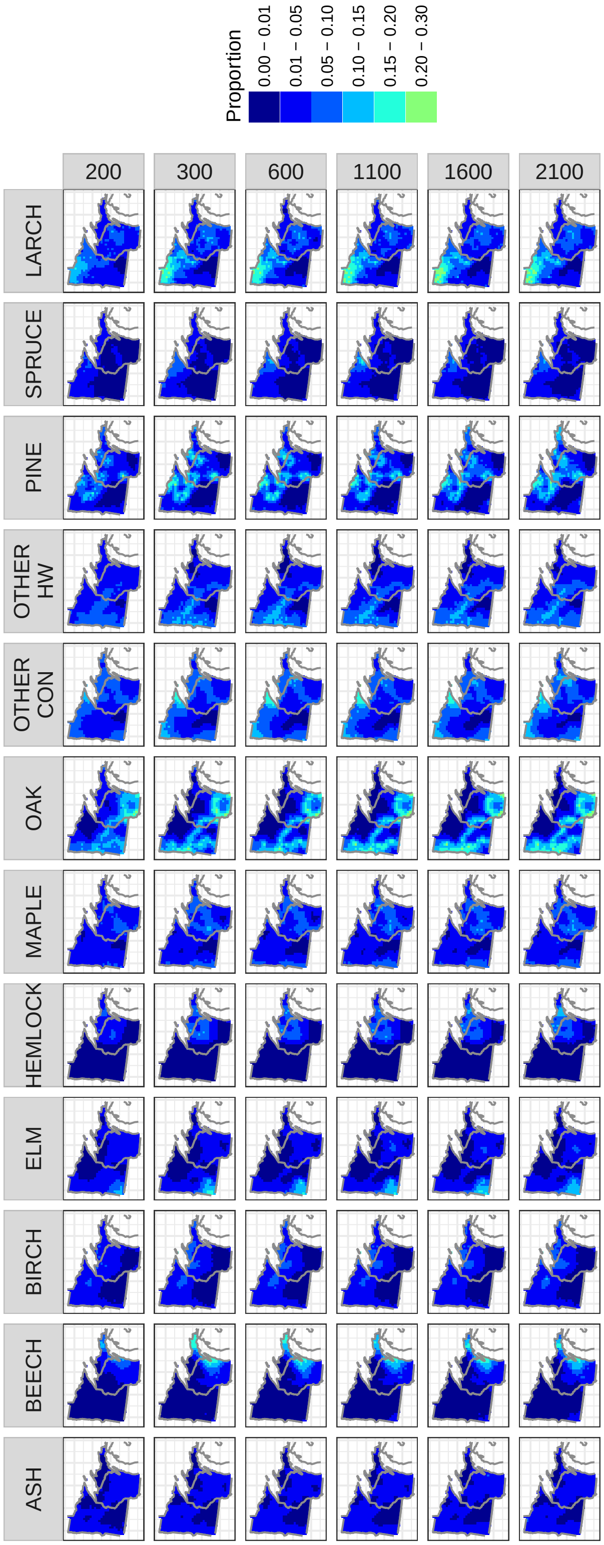

Figure S2: Standard deviation of the estimates of relative forest composition by taxon for a subset of time intervals.

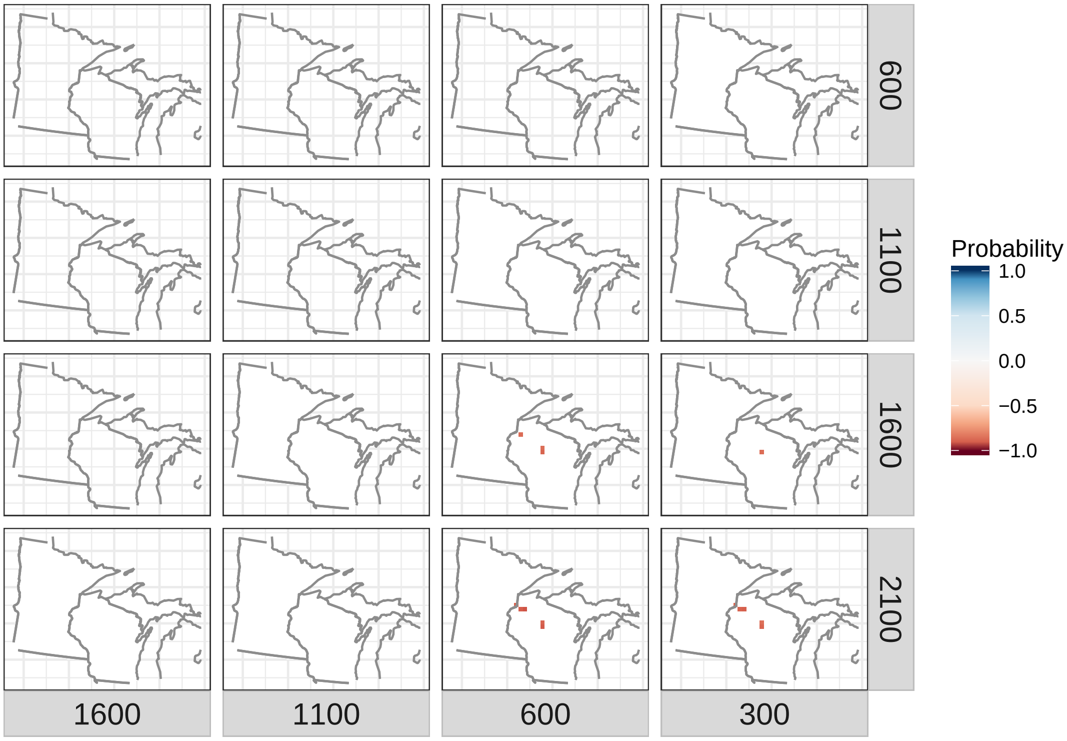

(a) Probability of significant change for ash.

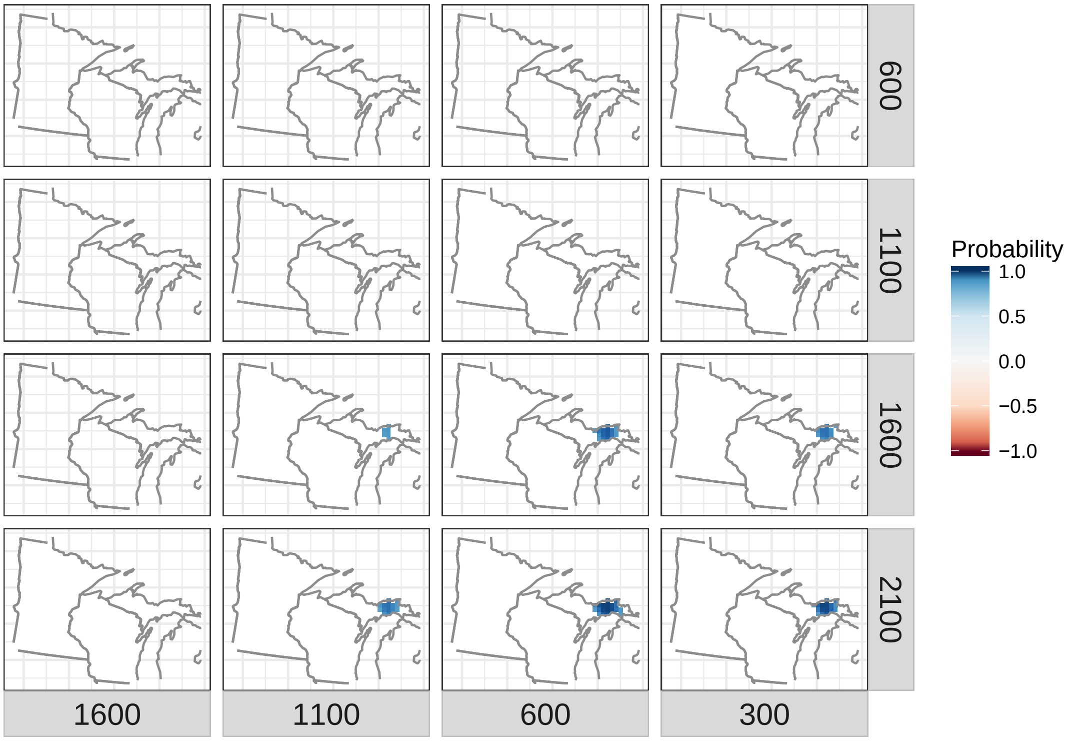

(b) Probability of significant change for beech.

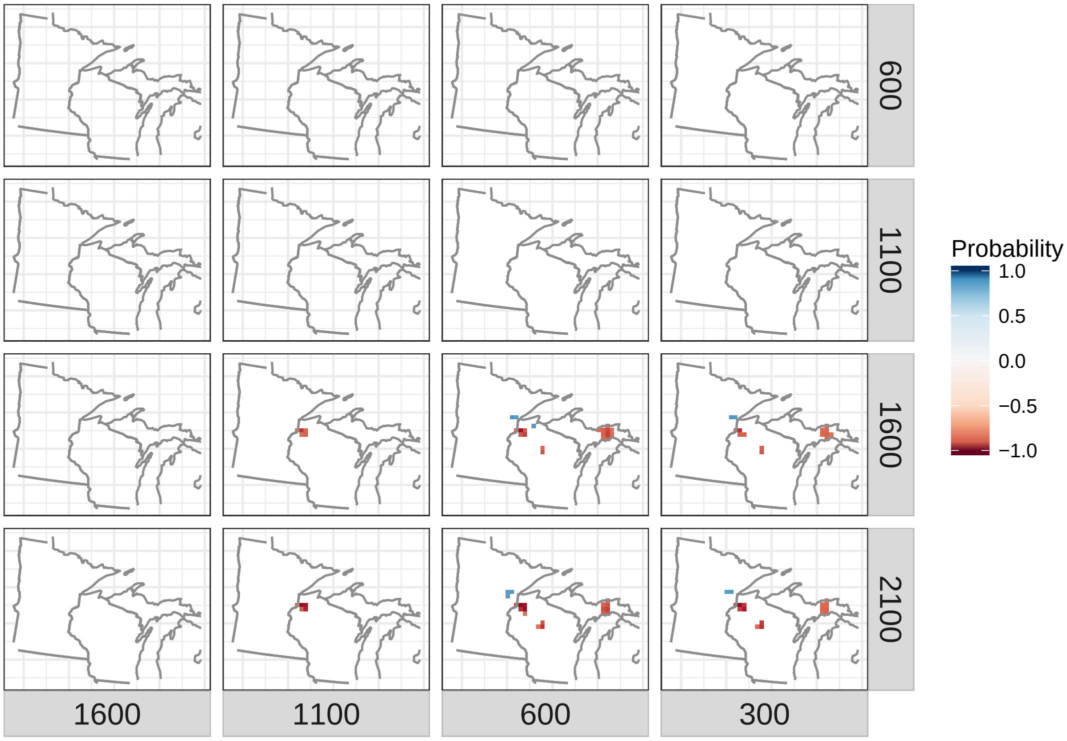

(c) Probability of significant change for birch.

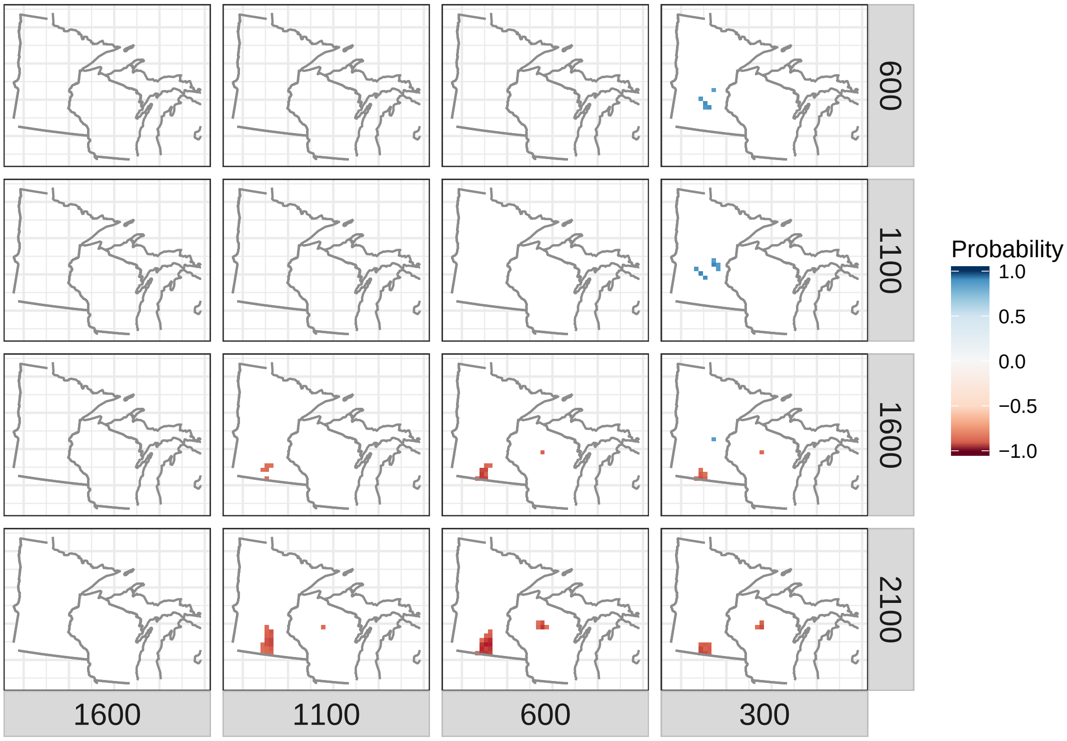

(d) Probability of significant change for elm.

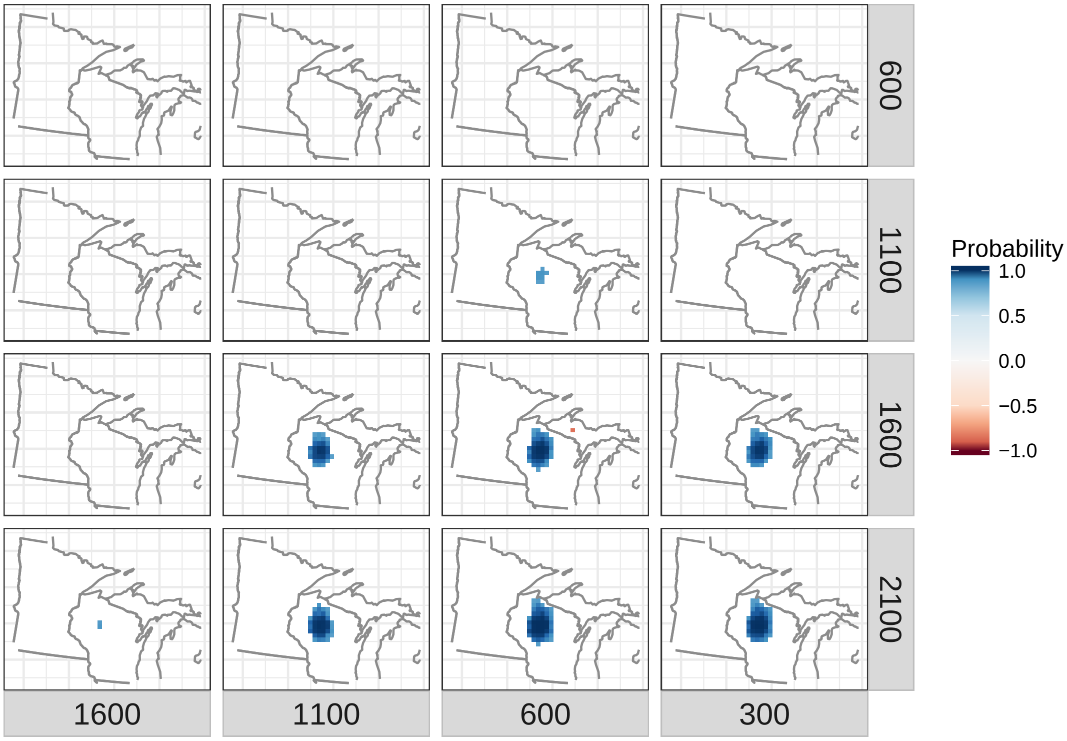

(e) Probability of significant change for hemlock.

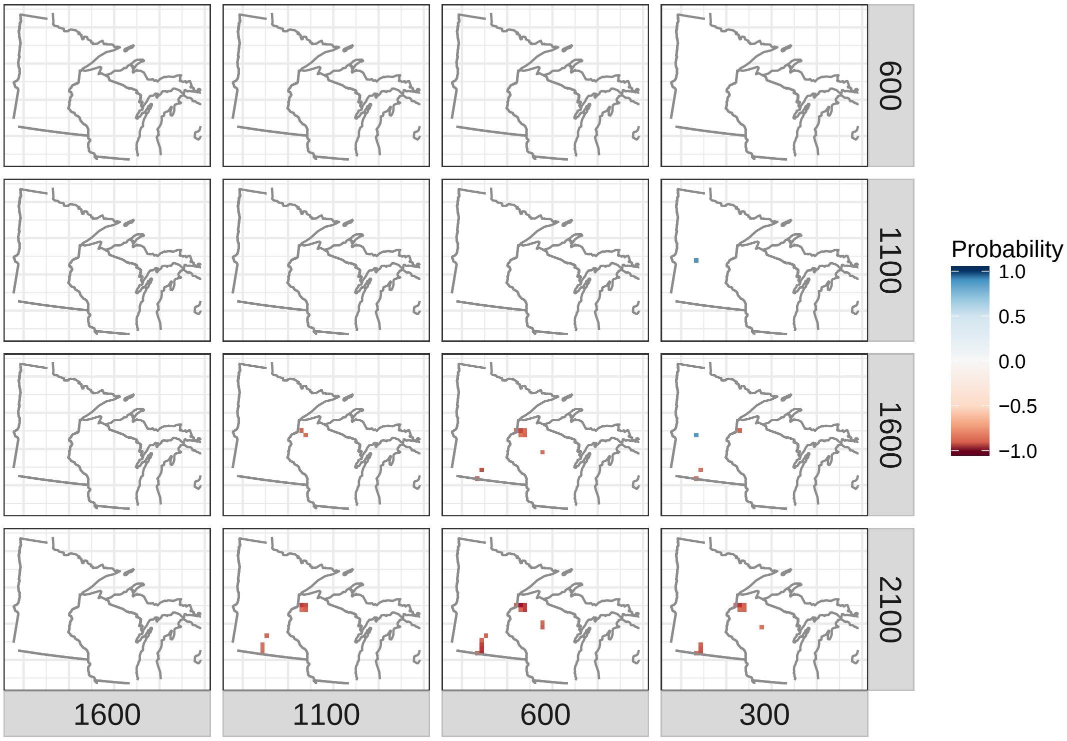

(f) Probability of significant change for maple.

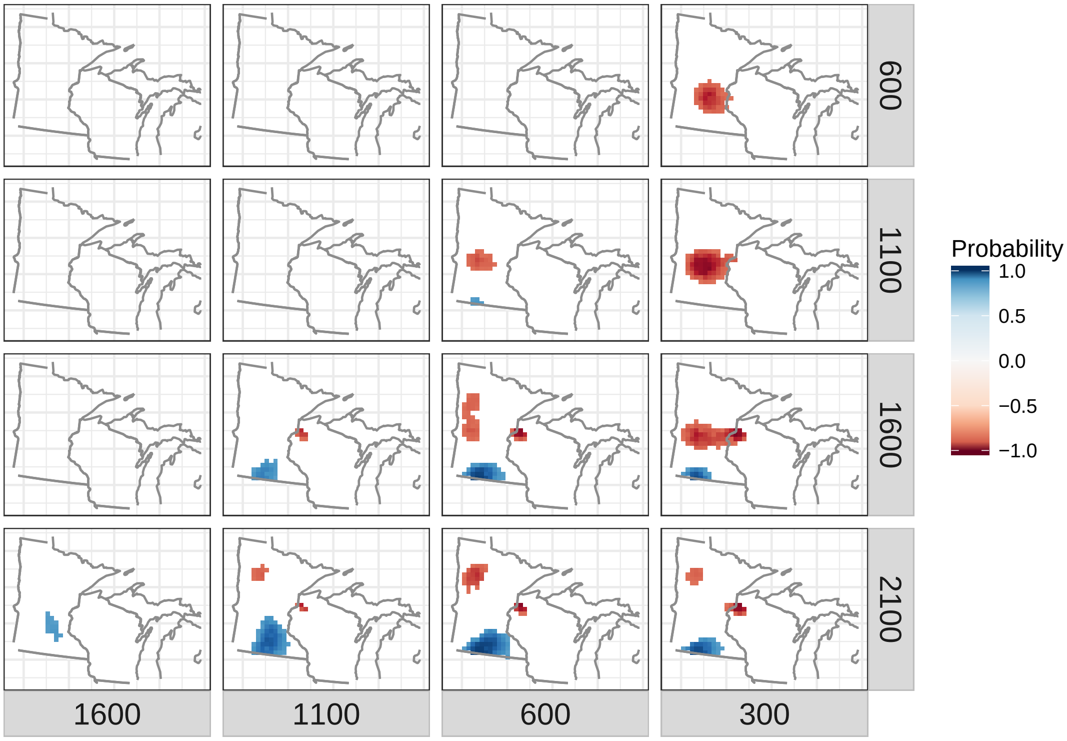

(g) Probability of significant change for oak.

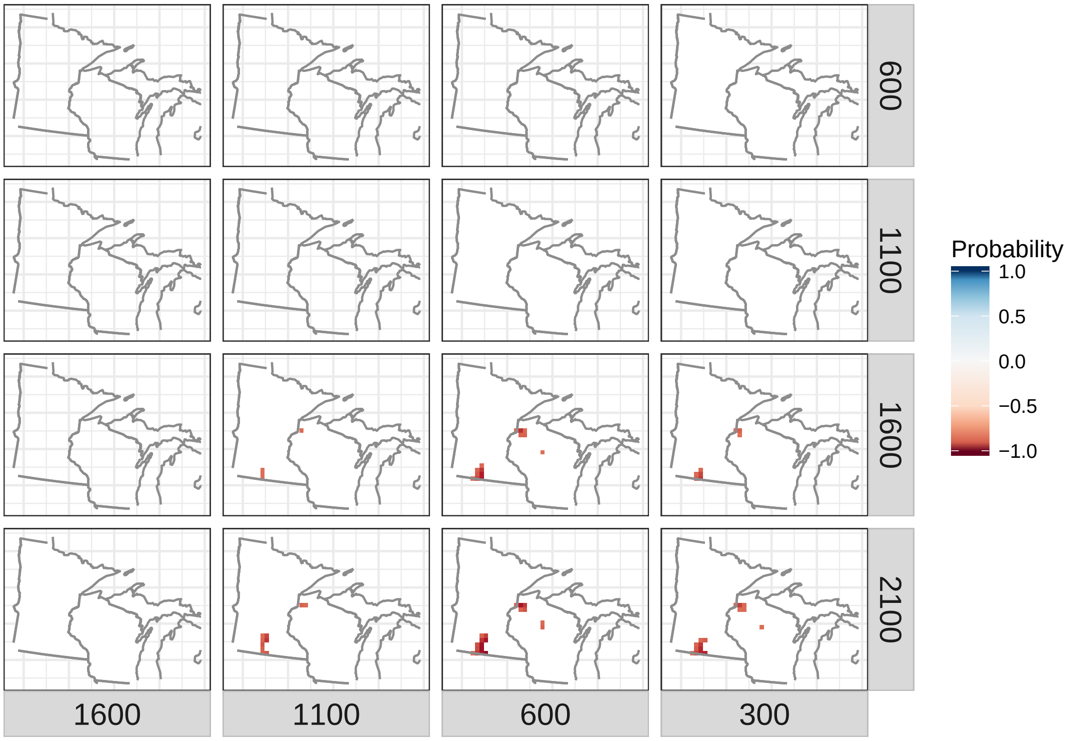

(h) Probability of significant change for other conifer.

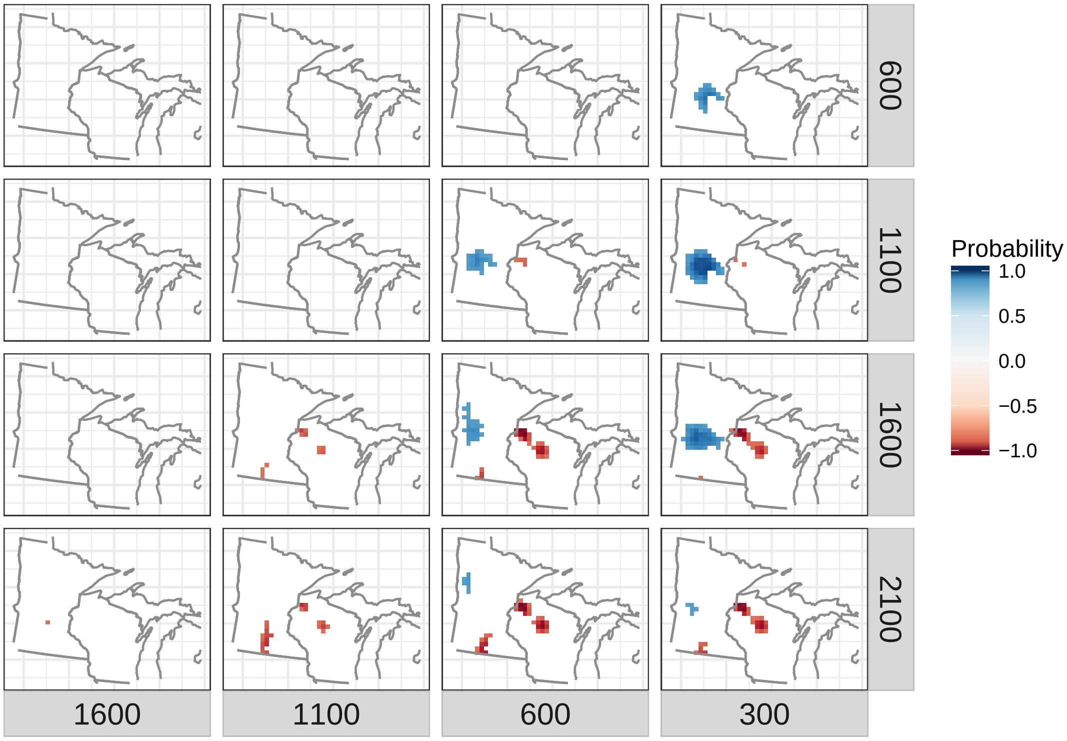

(i) Probability of significant change for other hardwood.

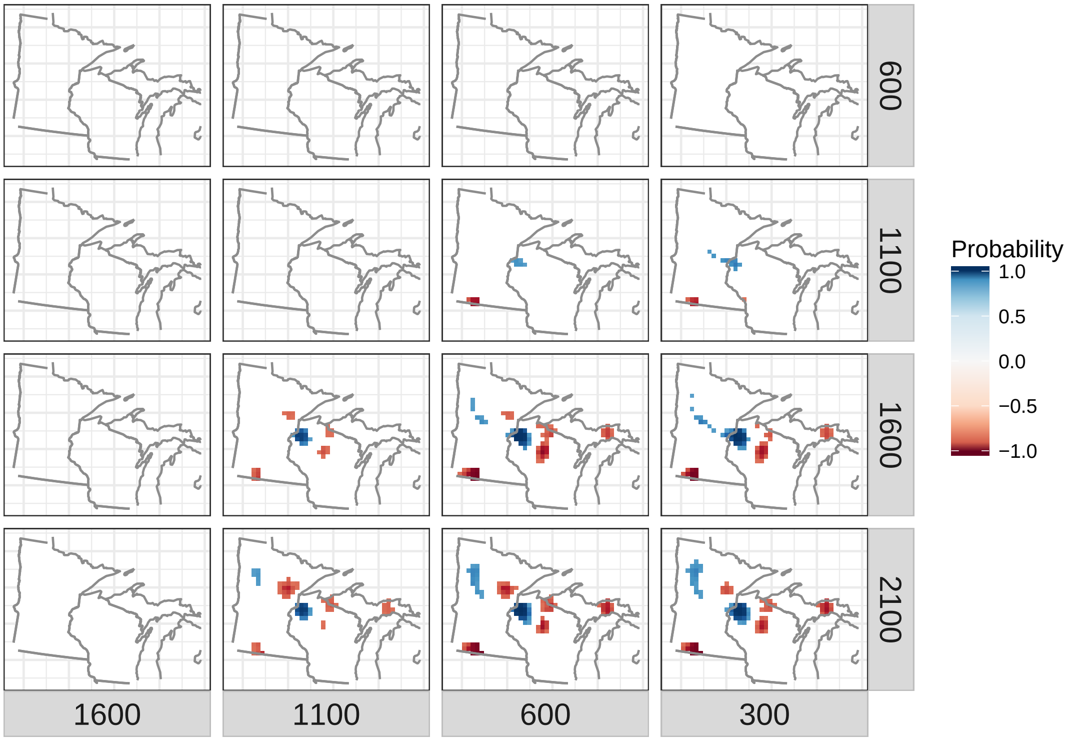

(j) Probability of significant change for pine.

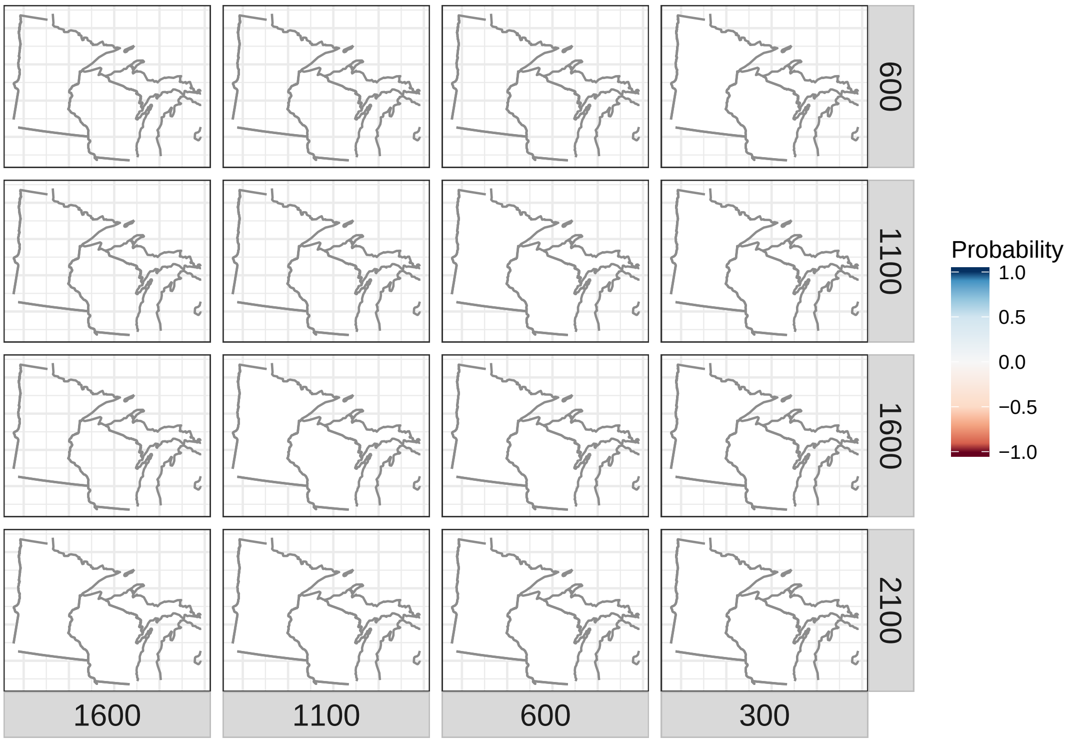

(k) Probability of significant change for spruce.

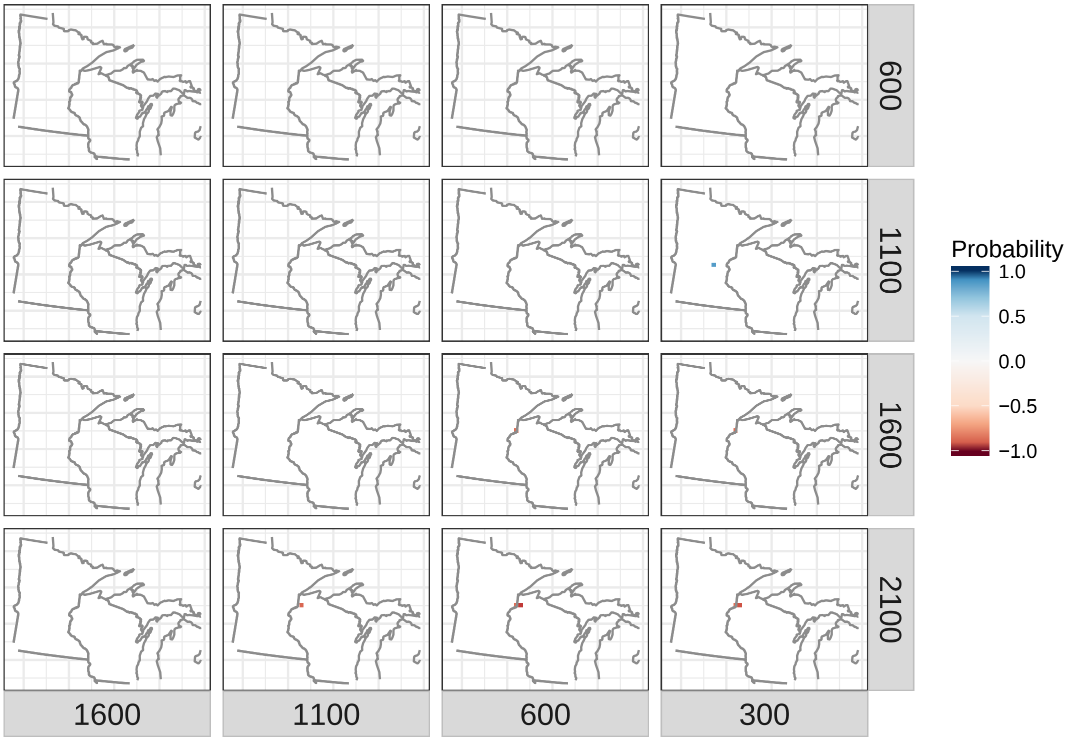

(l) Probability of significant change for tamarack.

Figure S3: Regions of significant change for the twelve considered taxa between time intervals. Blue indicates an increase in the more recent time while red indicates a decrease.

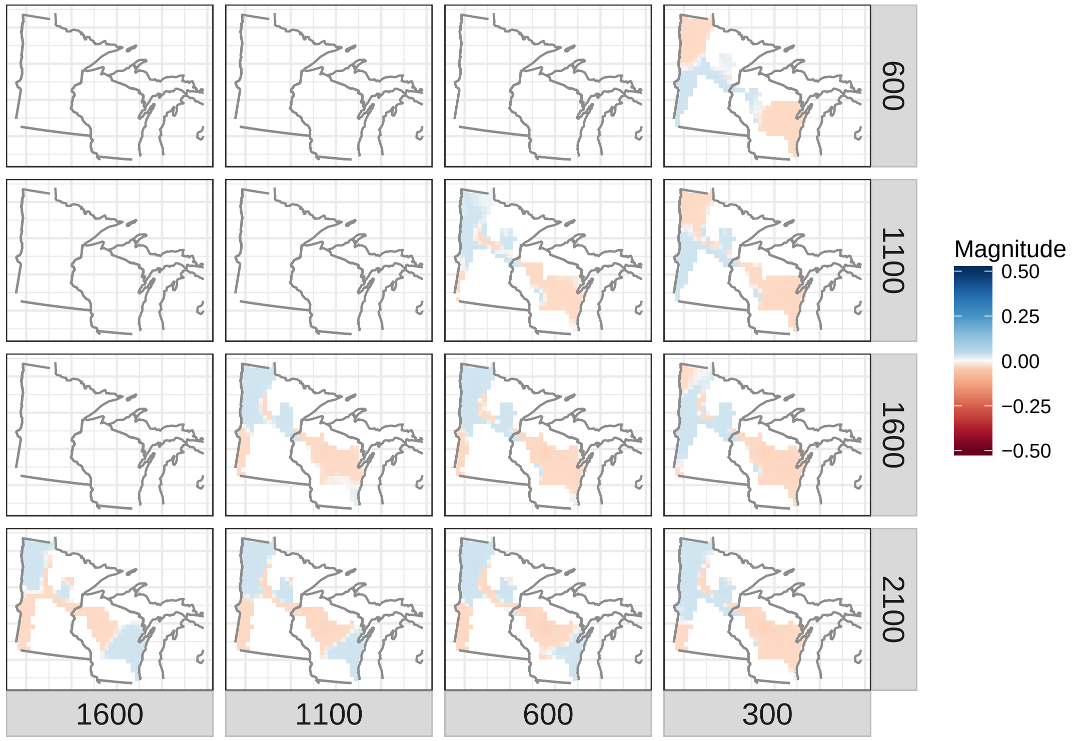

(a) Mean magnitude of change for ash.

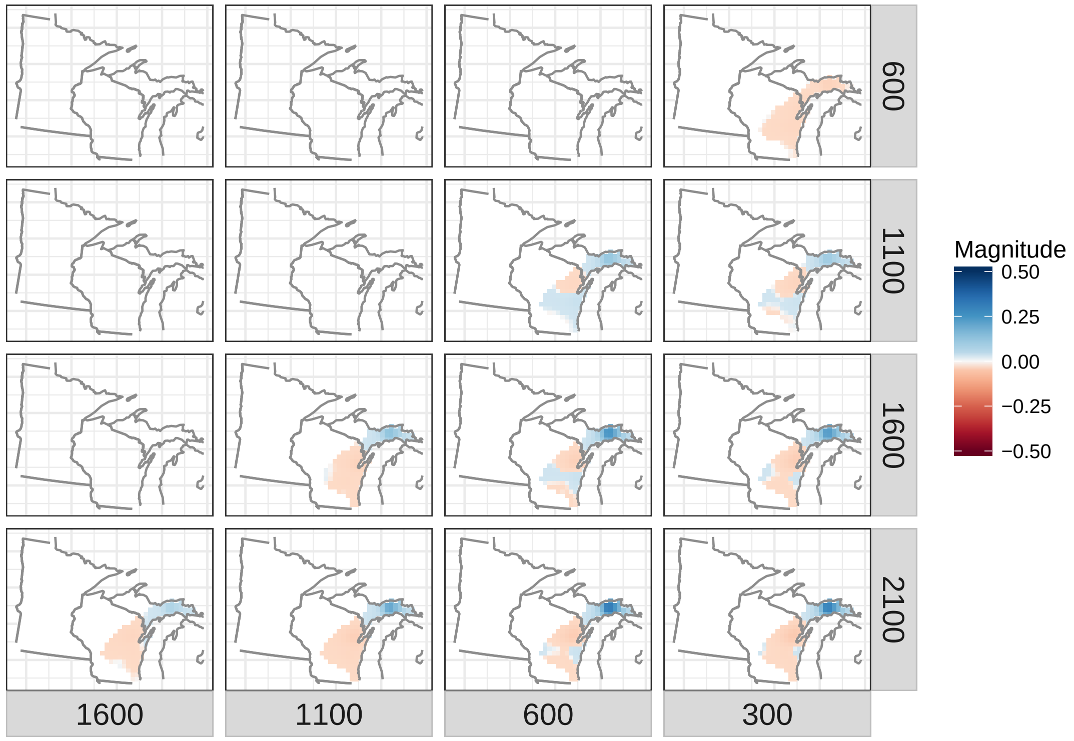

(b) Mean magnitude of change for beech.

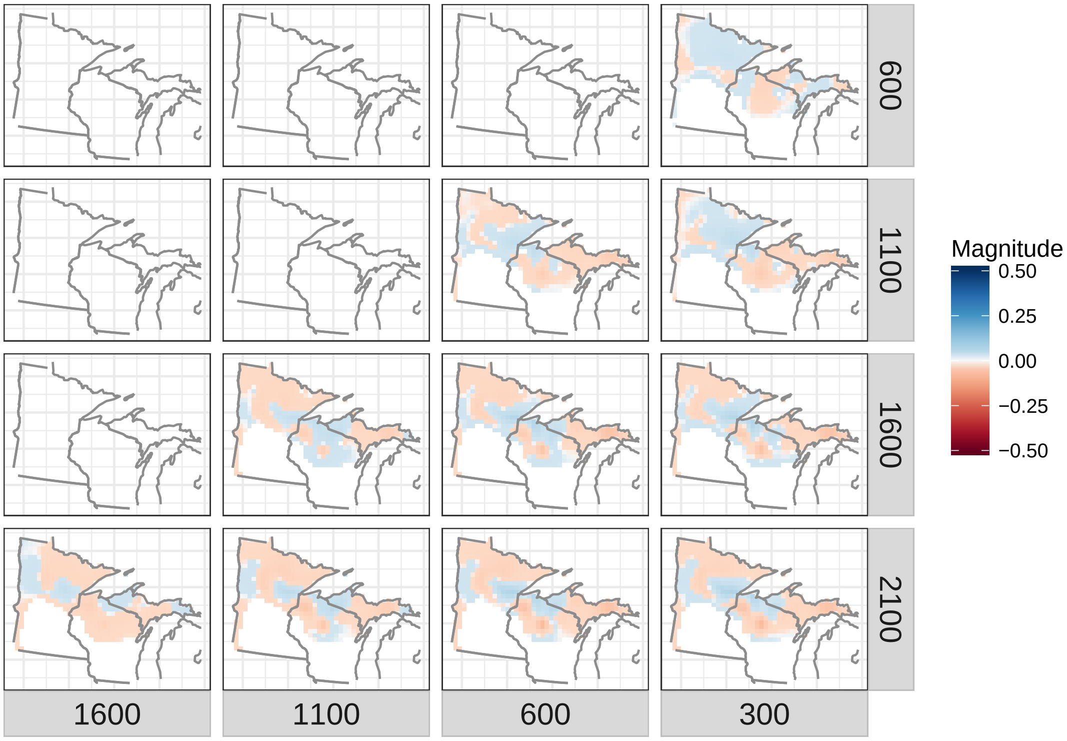

(c) Mean magnitude of change for birch.

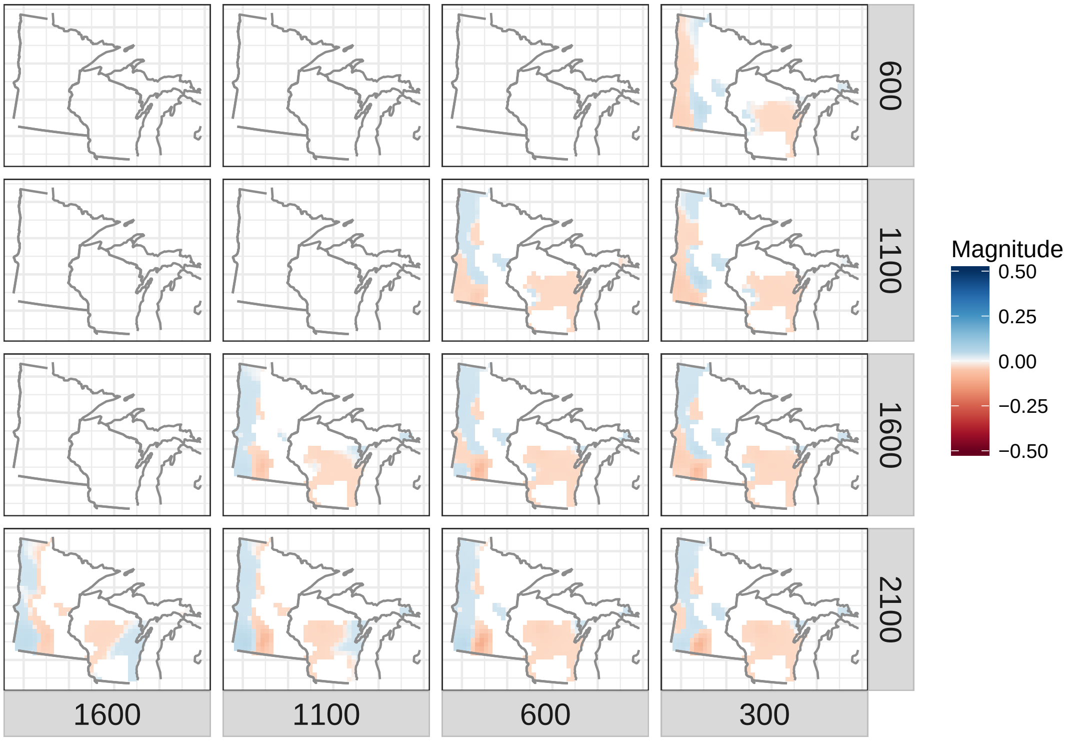

(d) Mean magnitude of change for elm.

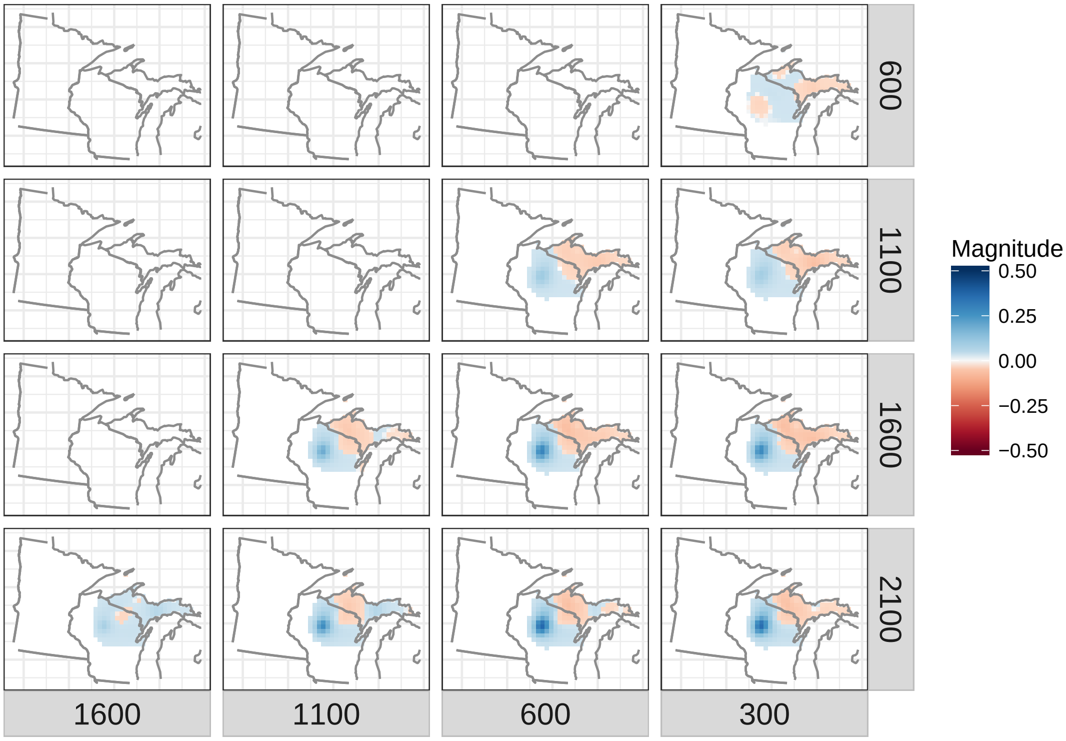

(e) Mean magnitude of change for hemlock.

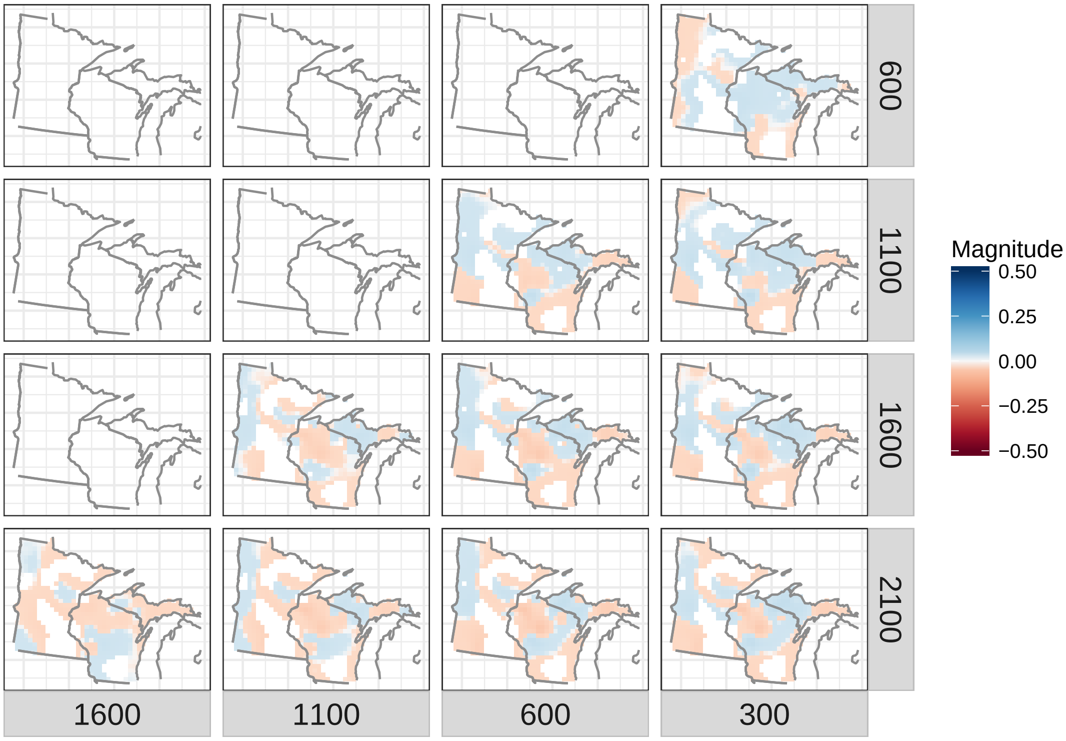

(f) Mean magnitude of change for maple.

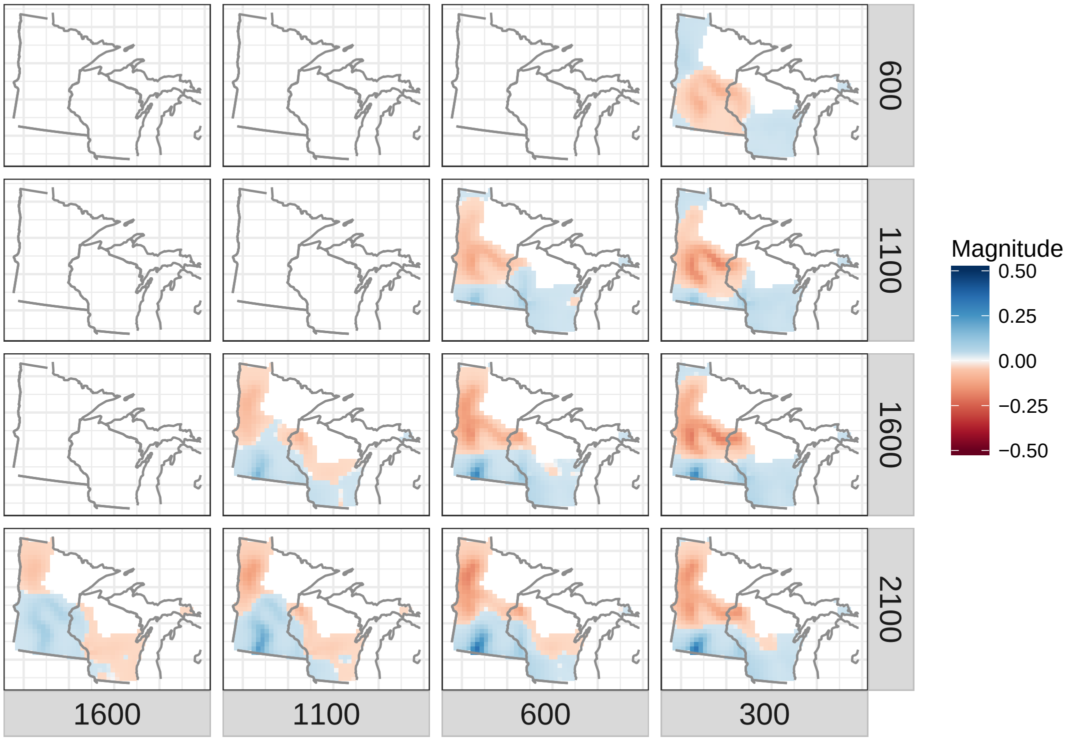

(g) Mean magnitude of change for oak.

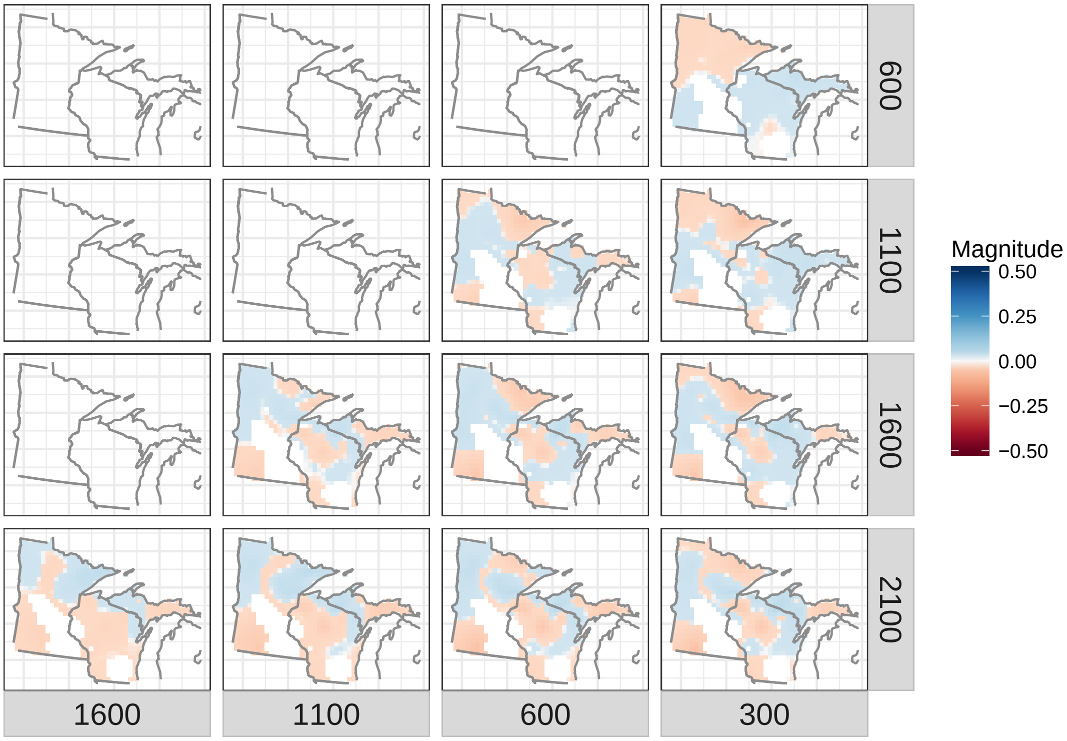

(h) Mean magnitude of change for other conifer.

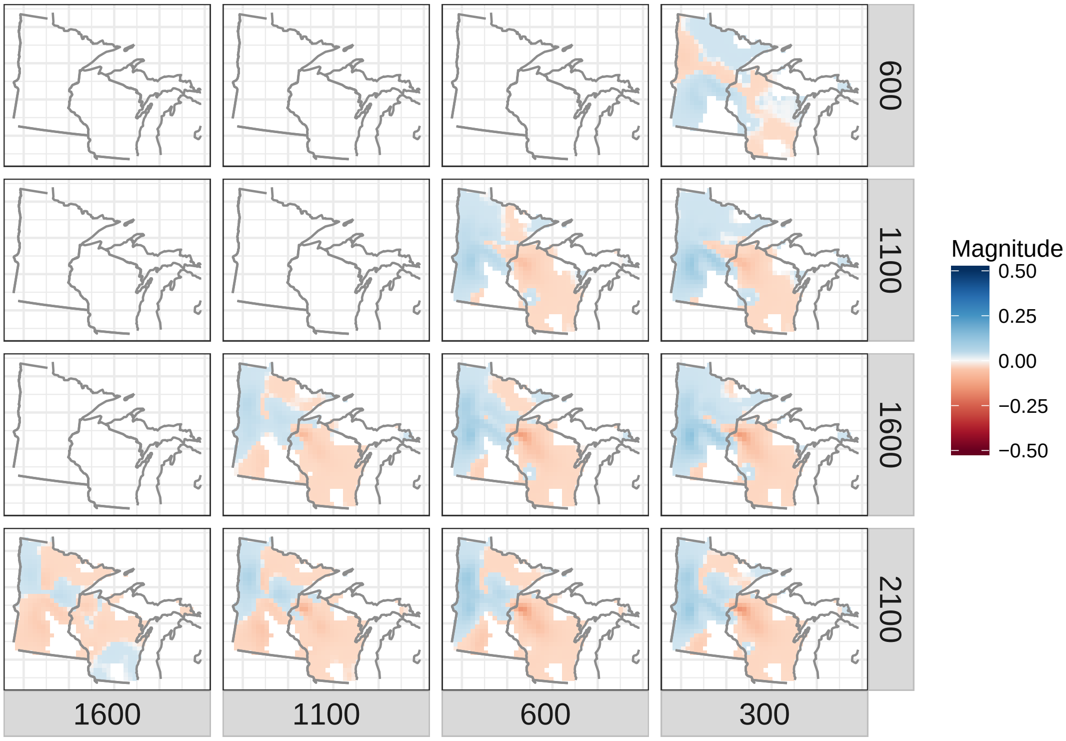

(i) Mean magnitude of change for other hardwood.

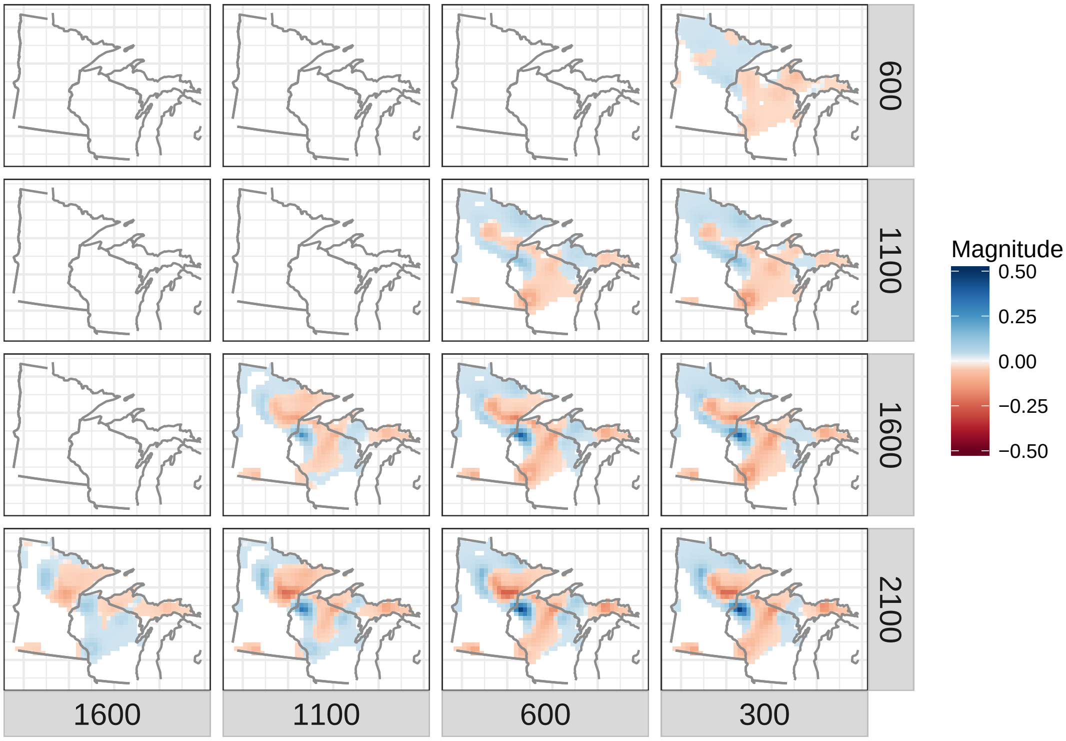

(j) Mean magnitude of change for pine.

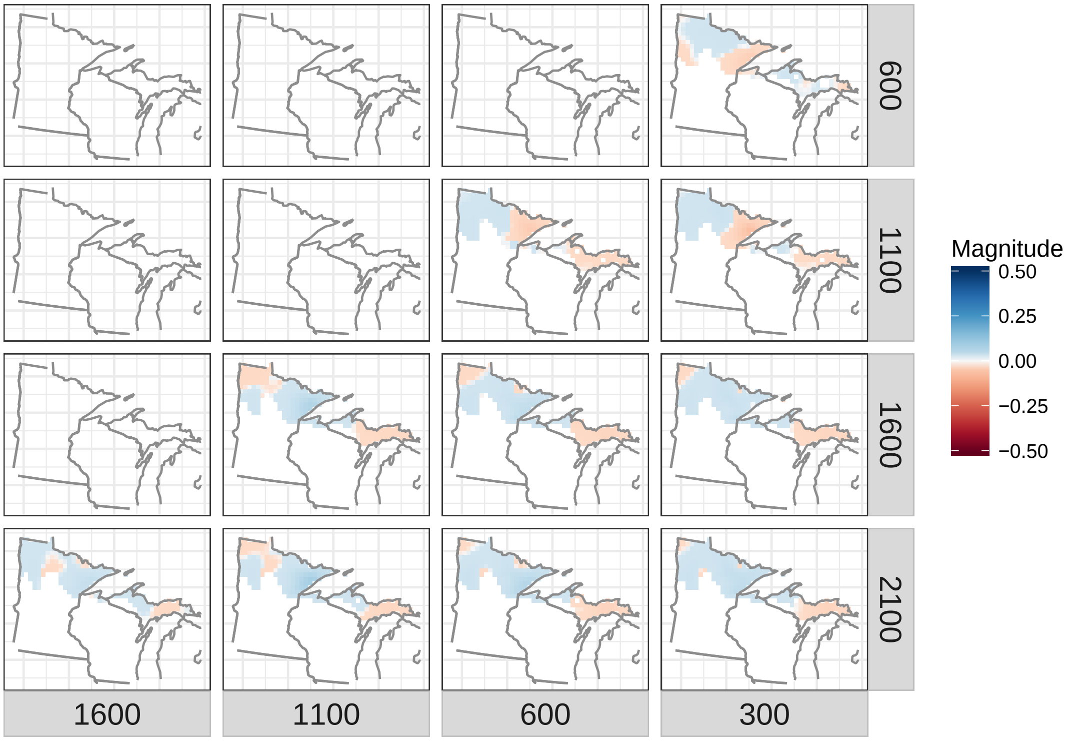

(k) Mean magnitude of change for spruce.

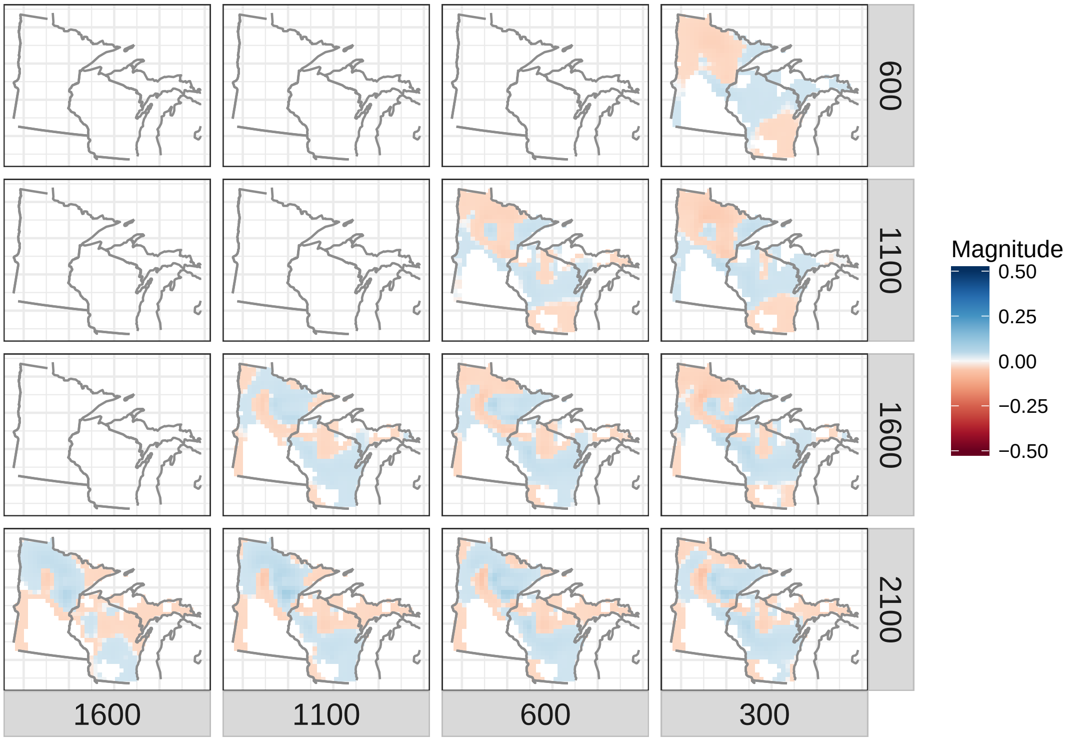

(l) Mean magnitude of change for tamarack.

Figure S4: Mean of differences of posterior estimates of forest composition for pairs of time intervals for twelve considered taxa. Blue indicates an increase in the more recent time while red indicates a decrease.

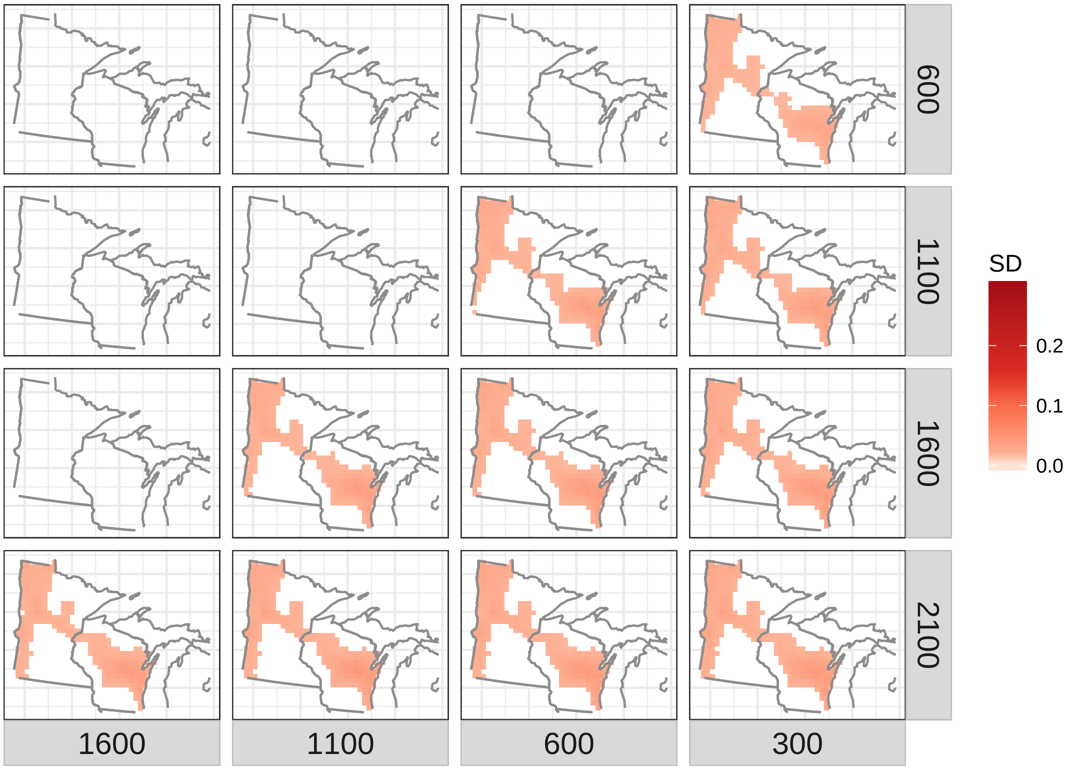

(a) Standard deviation of change for ash.

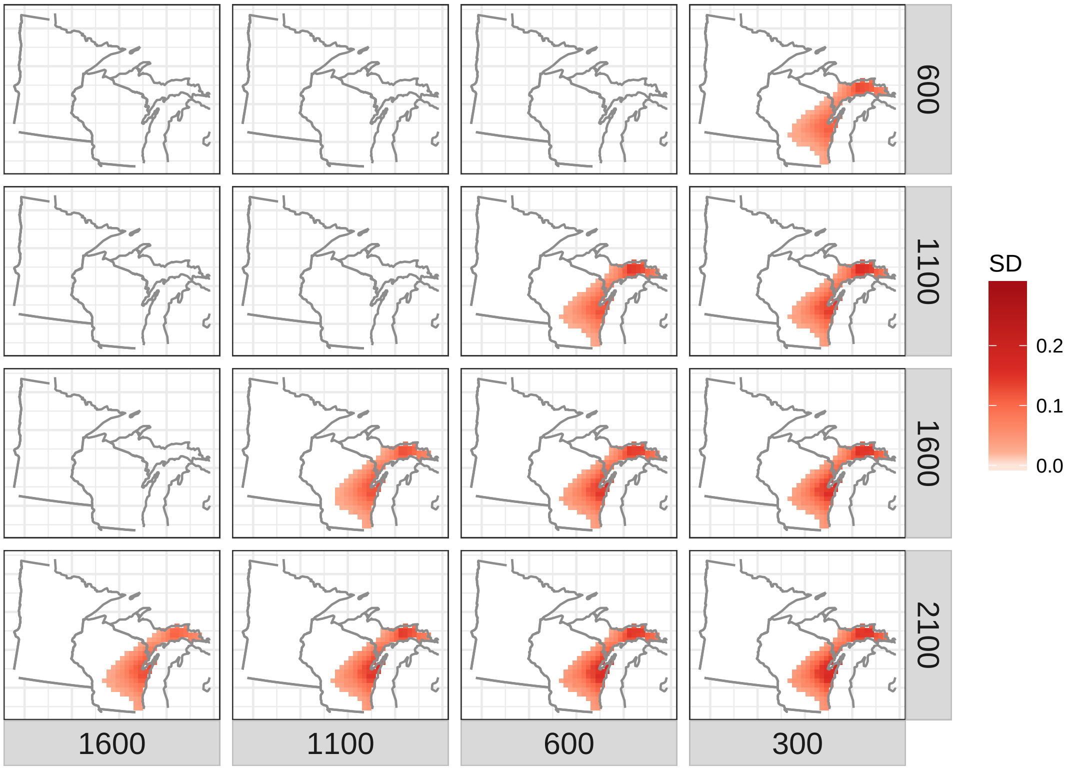

(b) Standard deviation of change for beech.

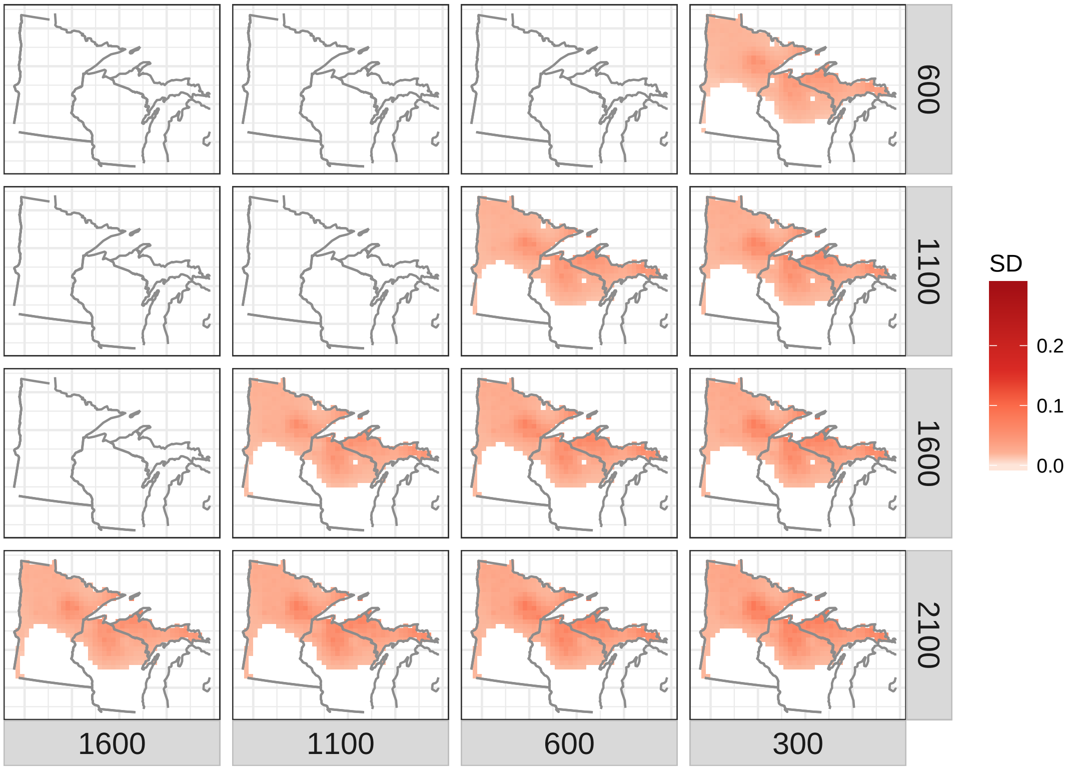

(c) Standard deviation of change for birch.

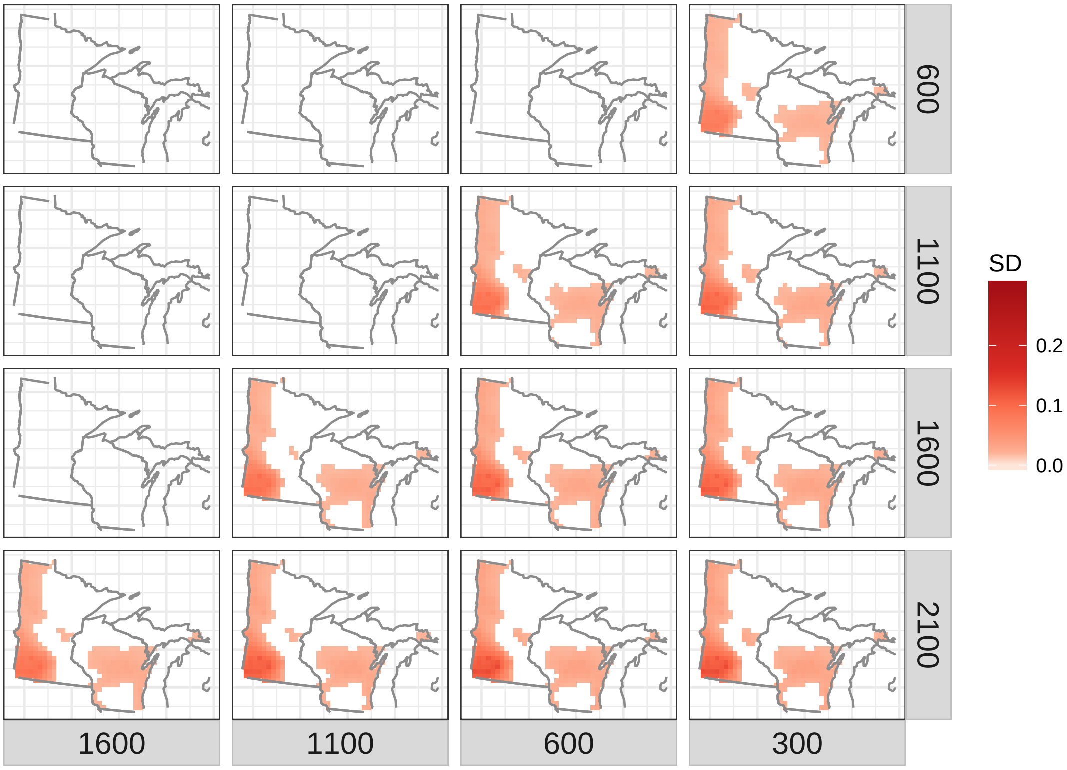

(d) Standard deviation of change for elm.

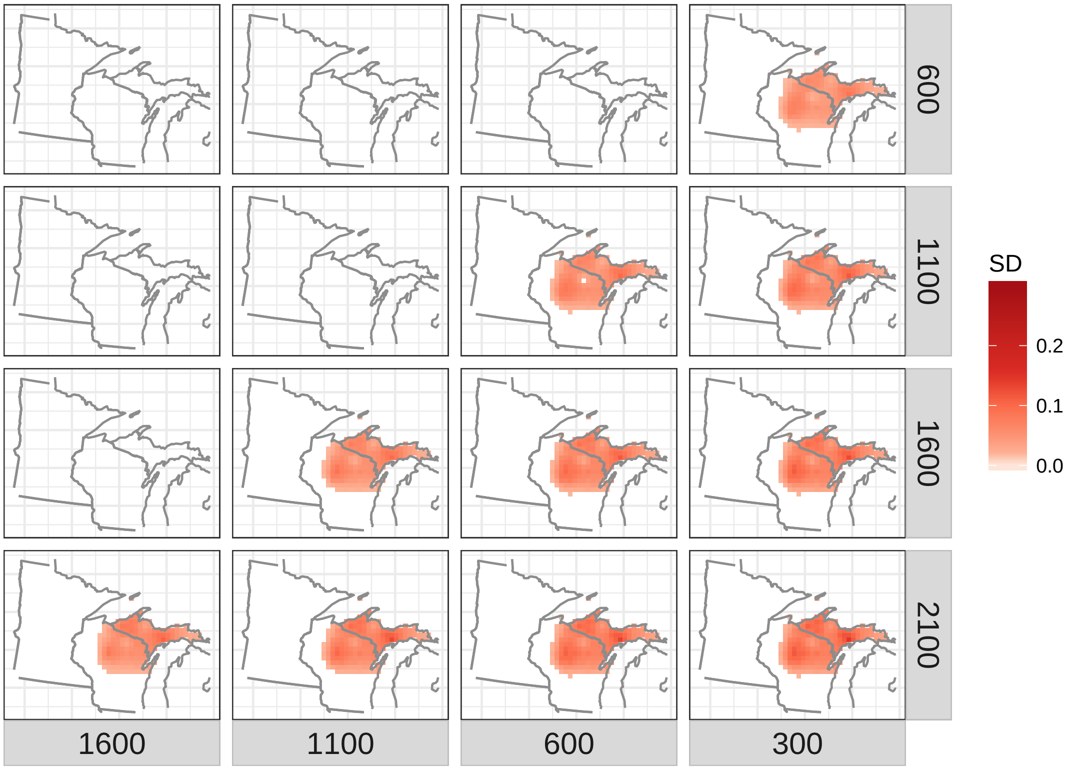

(e) Standard deviation of change for hemlock.

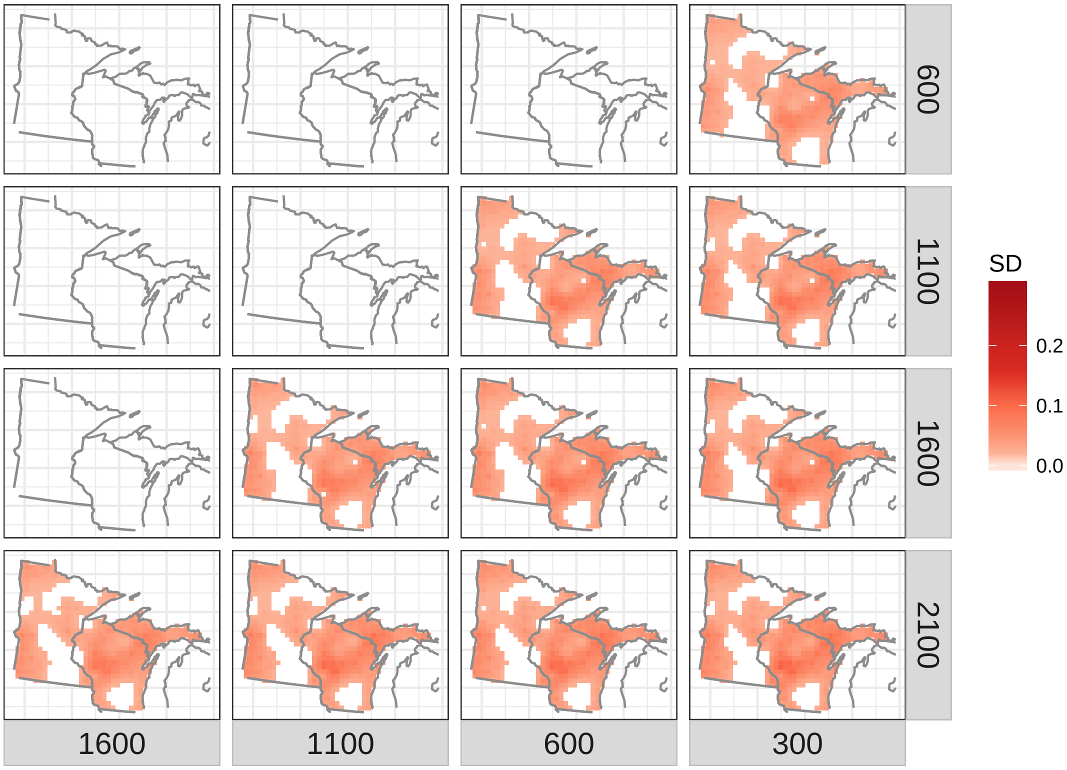

(f) Standard deviation of change for maple.

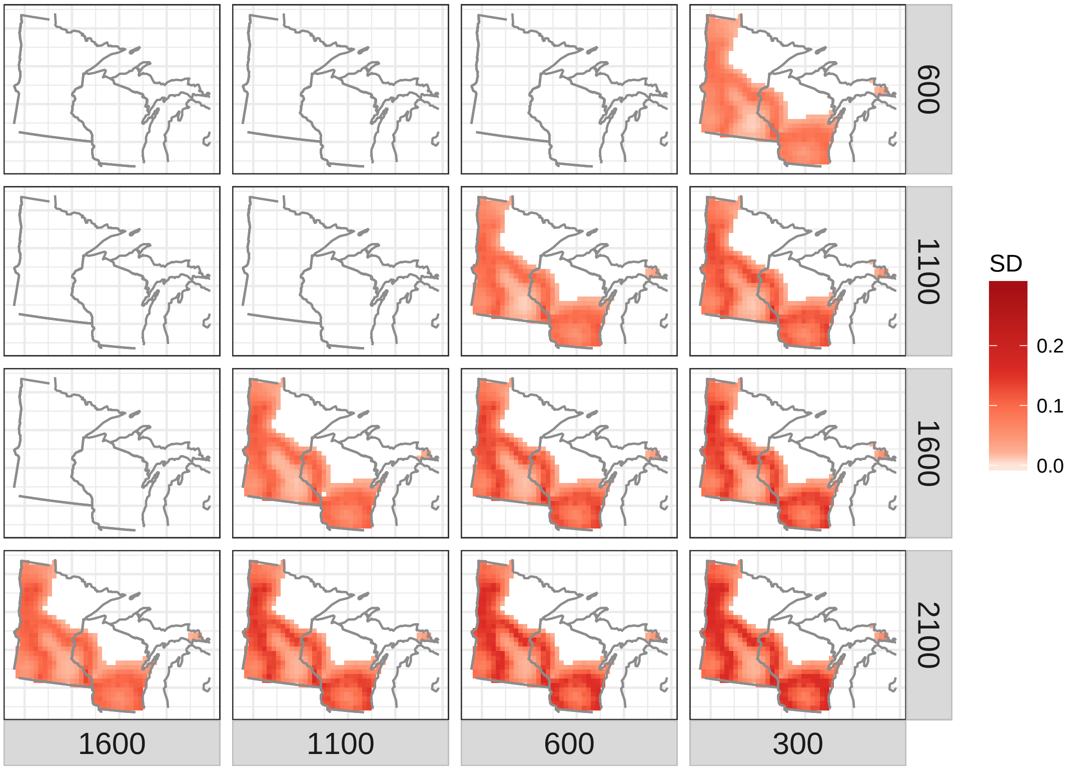

(g) Standard deviation of change for oak.

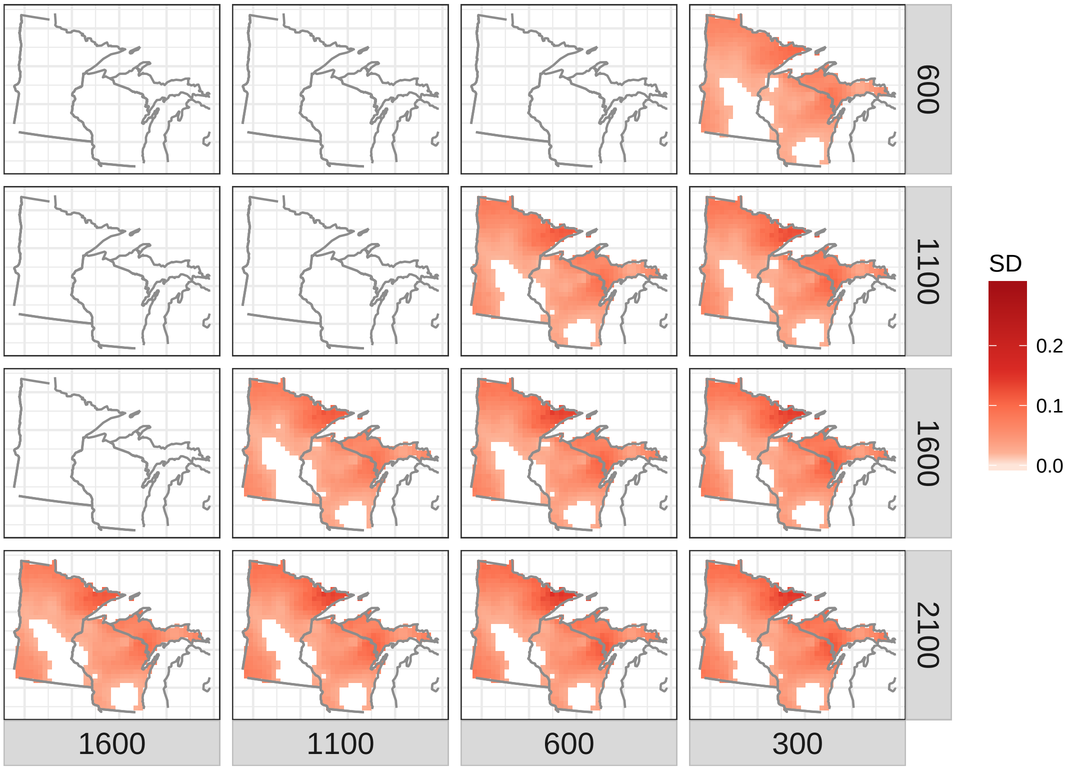

(h) Standard deviation of change for other conifer.

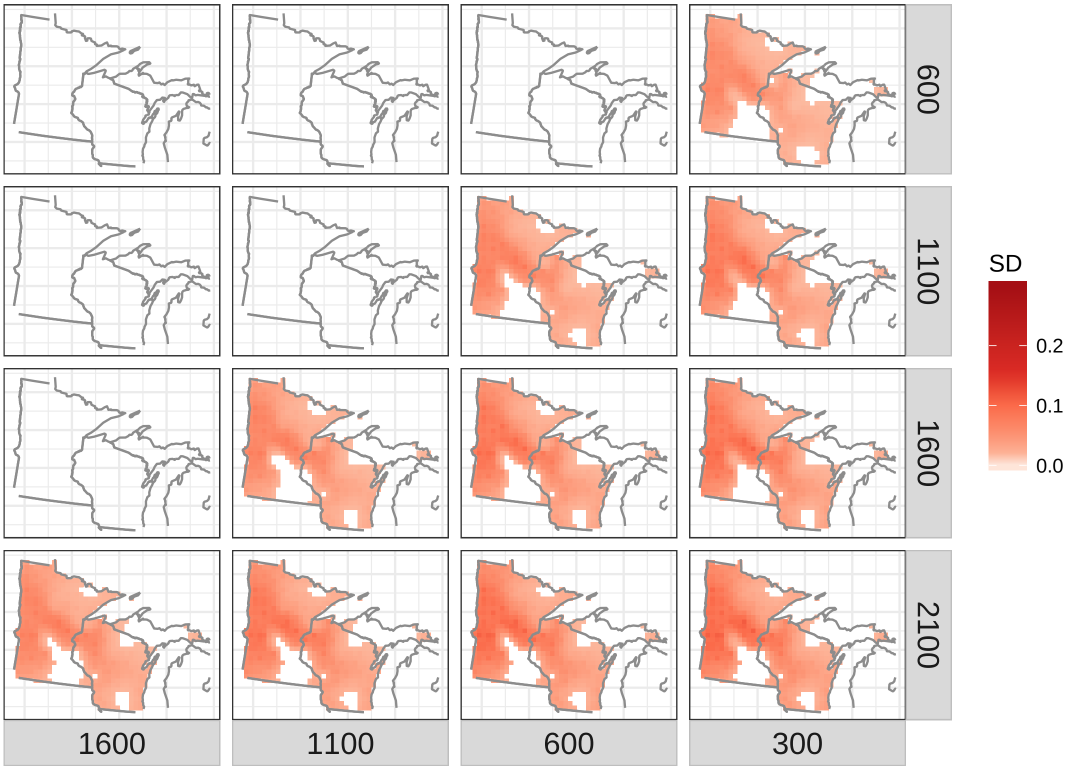

(i) Standard deviation of change for other hardwood.

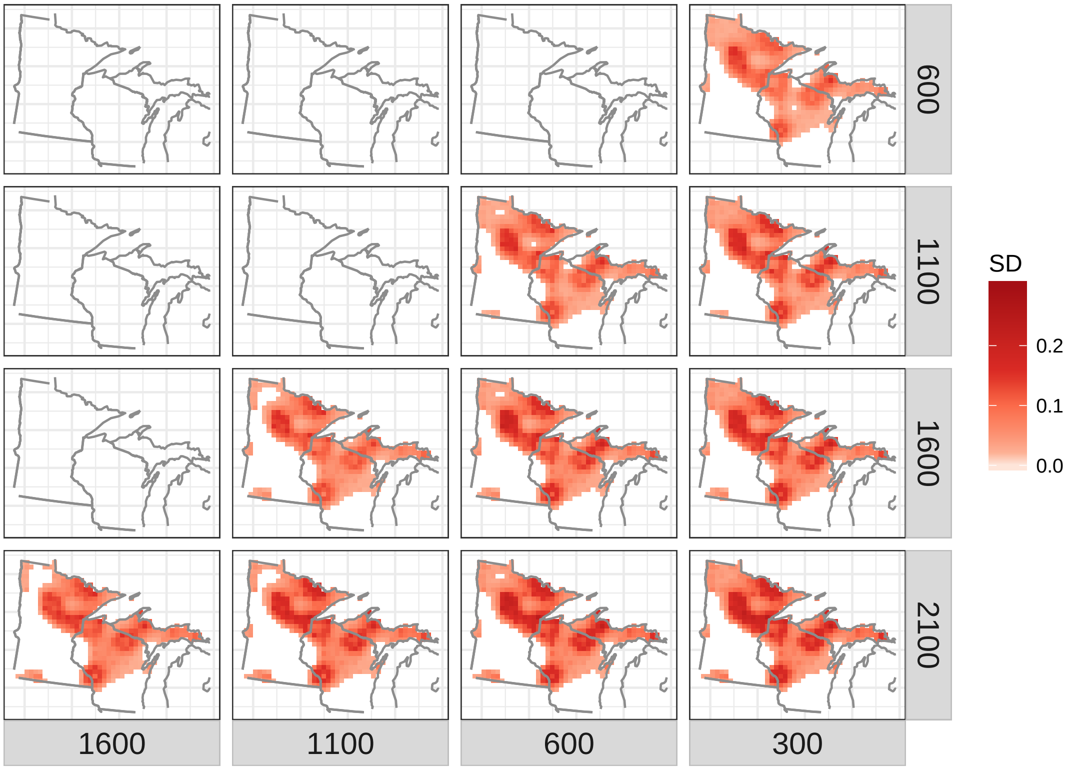

(j) Standard deviation of change for pine.

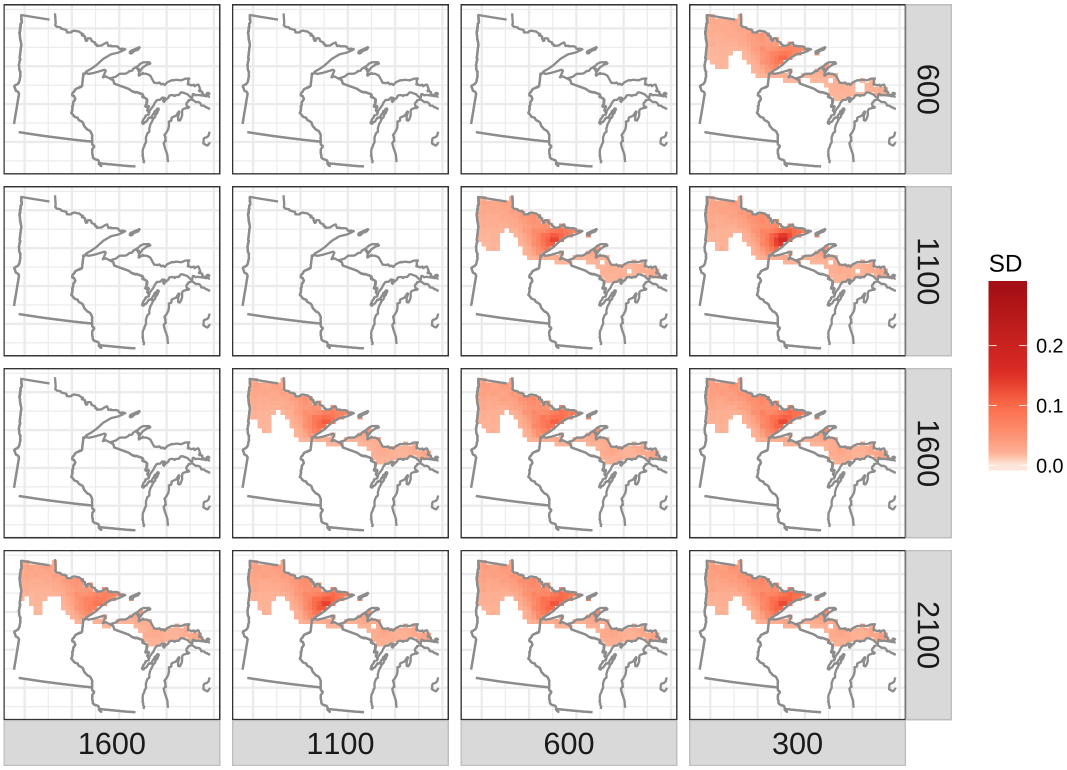

(k) Standard deviation of change for spruce.

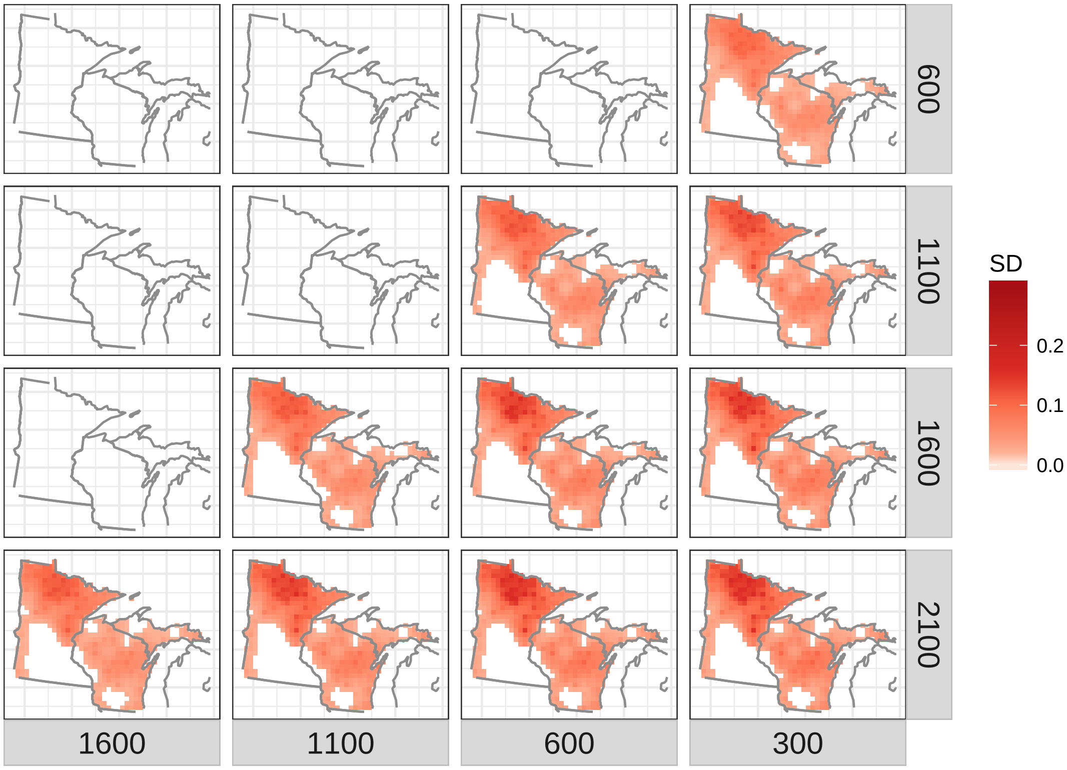

(l) Standard deviation of change for tamarack.

Figure S5: Standard deviation of differences of posterior estimates of forest composition for pairs of time intervals for twelve considered taxa.
